# Association of Exercise-Induced Pulmonary Hypertension and Echocardiographic Parameters of Cardiac Function: A Systematic Review and Meta-Analysis

**DOI:** 10.31083/RCM51027

**Published:** 2026-10-20

**Authors:** Xianrui Chen, Xiaoying Liu, Jian Wang, Qian Gao, Xinying Li, Wei Wang, Yishu Liu, Menglu Ouyang, Lin Huang, Bo Hu, Hui Yu, Chuting Wang, Yu Shi, Zhihui Wang, Guiyuan Han, Shuidong Feng, Yichong Li, Ke Peng

**Affiliations:** ^1^School of Public Health, University of South China, 421001 Hengyang, Hunan, China; ^2^National Clinical Research Center for Cardiovascular Diseases, Fuwai Hospital Chinese Academy of Medical Sciences, 518057 Shenzhen, Guangdong, China; ^3^Department of Respiratory Medicine, China-Japan Friendship Hospital, Capital Medical University, 100029 Beijing, China; ^4^Center for Clinical and Epidemiologic Research, Beijing Anzhen Hospital, Capital Medical University, 100029 Beijing, China; ^5^School of Public Health, Nanjing Medical University, 211166 Nanjing, Jiangsu, China; ^6^The George Institute for Global Health, Faculty of Medicine, University of New South Wales, Sydney, NSW 2031, Australia; ^7^Department of Rehabilitation Medicine, Shenzhen Prevention and Treatment Center for Occupational Diseases, 518020 Shenzhen, Guangdong, China; ^8^Department of Rehabilitation Medicine, Peking University Shenzhen Hospital, 518036 Shenzhen, Guangdong, China; ^9^Department of Rehabilitation Medicine, The 8th Affiliated Hospital, Sun Yat-Sen University, 518016 Shenzhen, Guangdong, China; ^10^Chinese Academy of Medical Sciences & Peking Union Medical College, 100006 Beijing, China; ^11^Clinical Research Center, Shenzhen Medical Academy of Research and Translation, 518017 Shenzhen, Guangdong, China

**Keywords:** exercise-induced pulmonary hypertension, cardiac function, meta-analysis, systematic review

## Abstract

**Introduction:**

Exercise-induced pulmonary hypertension (EIPH) is an abnormal hemodynamic response characterized by a mean pulmonary artery pressure/cardiac output (mPAP/CO) slope >3 mmHg/L/min between rest and exercise conditions. This systematic review and meta-analysis aimed to quantitatively synthesize existing evidence and investigate the association between EIPH and echocardiographic parameters of cardiac function.

**Methods:**

Electronic databases were searched through April 2025 for observational studies comparing cardiac function in individuals with EIPH and without EIPH. A random‐effects meta‐analysis model was used to pool the mean differences (MDs) and standard errors (SEs).

**Results:**

A total of 18 records met the inclusion criteria and were included in the meta-analysis. Compared with non-EIPH controls, individuals with EIPH exhibited a significantly attenuated cardiac output (CO) response during exercise (MD: –1.50 L/min; 95% confidence interval (CI): –2.45 to –0.54; *p* < 0.001) and markedly higher pulmonary vascular resistance (PVR) (MD: 1.30 wood units (WU); *p* < 0.001). Regarding left ventricular (LV) diastolic function, EIPH was associated with significantly lower e′ velocities and higher E/e′ ratios, with the E/e′ difference widening from rest (MD: 1.48) to peak exercise (MD: 2.46; both *p* < 0.001). In contrast, resting left ventricular ejection fraction (LVEF) and left ventricular end-diastolic diameter (LVEDD) did not differ significantly between groups.

**Conclusions:**

EIPH represents an early pathological phenotype characterized by impaired cardiac pumping reserve and subclinical biventricular dysfunction, particularly occult LV diastolic impairment. These findings highlight EIPH as a critical window for detecting subclinical heart failure. Future research should prioritize investigation into disease progression in individuals with EIPH and evaluate whether early intervention can prevent progression to overt cardiovascular failure.

**The PROSPERO Registration:**

CRD42024506532, https://www.crd.york.ac.uk/PROSPERO/view/CRD42024506532.

## 1. Introduction

Exercise-induced pulmonary hypertension (EIPH) is a pathological hemodynamic phenotype defined by a mean pulmonary artery pressure/cardiac output (mPAP/CO) slope >3 mmHg/L/min at peak exercise [1,2,3]. In individuals without EIPH, the pulmonary vasculature accommodates increased CO by recruiting non-perfused capillaries and promoting vasodilation, thereby maintaining a low-resistance circuit with a physiological mPAP/CO slope (≤3 mmHg/L/min) and stable or decreased total pulmonary resistance (TPR) [2,4,5,6]. In contrast, individuals with EIPH exhibit impaired vascular reserve and fail to sufficiently reduce pulmonary vascular resistance (PVR) as CO rises during exercise, leading to an exaggerated increase in mPAP [7,8,9,10,11]. The abnormally elevated mPAP and TPR during exercise impose significant mechanical strain on the cardiovascular system, thereby increasing the risk of adverse cardiovascular outcomes [12,13,14].

The precise pathogenesis of EIPH remains incompletely understood, as exercise-induced changes in pulmonary hemodynamics are jointly determined by CO, PVR, and left atrial pressure (LAP) [15,16,17,18]. Abnormally elevated PVR during exercise, deemed an early hemodynamic marker of left ventricular (LV) diastolic dysfunction, has been identified as a potential mechanism underlying the pathogenesis of EIPH in previous studies [19,20]. Chronic exposure to elevated pulmonary artery pressure (PAP) may trigger a transition from reversible vascular distension to permanent structural remodeling [12], potentially leading to a progressive decline in the capacity of the pulmonary vessels to accommodate increased pulmonary blood flow, and eventually resulting in resting pulmonary hypertension. The gold-standard measurement, right heart catheterization (RHC), allows direct assessment of hemodynamic shifts. However, RHC is challenging to perform owing to the invasive nature and technical complexity of the procedure under exercise conditions. Thus, there remains an urgent need for validated non-invasive imaging diagnostic tools suitable for clinical practice and public health screening.

Current literature reveals that EIPH is increasingly recognized as a predictor of persistent pulmonary hypertension (PH) and cardiovascular diseases [14,21]. Older adults appear to be more susceptible to EIPH, likely due to age-related increases in mean arterial pressure (MAP), PVR, and systemic vascular resistance (SVR) [22]. Furthermore, EIPH also serves as a biomarker in various pathological states, including *bone morphogenetic protein receptor type 2* gene mutations, systemic sclerosis, left heart conditions, chronic lung diseases, and chronic thromboembolic pulmonary disease [14]. EIPH is also associated with reduced exercise capacity and early indication of cardiac dysfunction, characterized by decreased CO, reduced mitral annular velocity (e′), and an elevated early ratio of transmitral flow velocity to early mitral annular velocity (E/e′), which often precedes the onset of PH [23,24,25]. These early impairments in right ventricular (RV) pulmonary vascular coupling suggest that EIPH may represent a critical window for intervention before irreversible cardiac structural remodeling occurs.

Although numerous studies have explored the hemodynamic characteristics of EIPH, available data regarding the effect of EIPH on cardiac function remain heterogeneous and fragmented. Therefore, this systematic review and meta-analysis aimed to provide a rigorous evaluation of existing evidence on the association between EIPH and cardiac function. Specifically, we analyzed: (1) pulmonary hemodynamic parameters, including mPAP, PVR, and CO; (2) LV diastolic function, including the early diastolic filling velocity to late diastolic filling velocity ratio (E/A ratio), mitral annular early diastolic velocity (e′ wave velocity), early transmitral flow velocity to early mitral annular velocity ratio (E/e′ ratio); (3) LV systolic function, including left ventricular ejection fraction (LVEF), left ventricular end-diastolic diameter (LVEDD), left ventricular end-systolic diameter (LVESD), and stroke volume (SV). By synthesizing the existing evidence, this review aimed to elucidate the structural and functional cardiac alterations associated with EIPH and to inform future clinical management, research priorities, and public health strategies.

## 2. Methods

### 2.1 Literature Search Strategy

This systematic review and meta-analysis were performed in accordance with the Preferred Reporting Items for Systematic Reviews and Meta-Analyses (PRISMA) statement and checklist [26]. The protocol was registered with PROSPERO (CRD42024506532). We systematically searched PubMed, Embase, and Web of Science databases for articles published up to April 2025. The search strategy included the following keywords and related terms: “Pulmonary Hypertension”, “Pulmonary Arterial Hypertension (PAH)”, “PH”, “Pulmonary Vascular”, “Exercise-Induced”, “Exercise”, “Physical”, “Exercise Test”, “Exercise Tolerance”, “Ventricular Function”, “Left Ventricular Dysfunction”, “Heart Function Tests”, and “Cardiovascular Diseases”. The updated search was conducted on 30 April 2025 (**Supplementary Table 1**).

### 2.2 Inclusion and Exclusion Criteria

Studies that met the following criteria were included: (i) published as full-text articles in an English-language peer-reviewed journal; (ii) involved adults aged ≥18 years; (iii) used an observational study design (cross-sectional, case–control, or cohort); (iv) investigated individuals with EIPH and reported at least one quantitative indicator of cardiac function; (v) grey literature, including conference abstracts and posters, was eligible for inclusion if it met all predefined criteria and provided sufficient data for extraction and analysis.

Studies were excluded if: (i) the population comprised individuals with exercise-induced pulmonary hemodynamic changes driven by physiological adaptation (*e*.*g*., professional athletes) or distinct disease-specific mechanisms (*e*.*g*., HIV-associated PH); (ii) a clear definition of EIPH was not provided; or (iii) data were insufficient to extract or analyze cardiac function outcomes in individuals with EIPH.

### 2.3 Outcomes

The primary outcome of interest was the difference in cardiac function indicators, including the following parameters: mPAP, PVR, CO, early diastolic filling velocity to late diastolic filling velocity ratio (E/A ratio), mitral annular early diastolic velocity (e′ wave velocity), early transmitral flow velocity to early mitral annular velocity ratio (E/e′ ratio), LVEF, LVEDD, LVESD, and SV.

### 2.4 Full-Text Assessment and Data Extraction

After removing duplicates, one reviewer (CXR) screened the titles and keywords for all retrieved articles, followed by independent abstract screening by two reviewers (CXR and WJ) to identify potentially eligible studies. Studies that compared cardiac function indicators between individuals with EIPH and non-EIPH controls were considered eligible. The same two reviewers then independently assessed the full-text articles to confirm eligibility. Discrepancies were resolved through discussion with a third reviewer (LXY). From each eligible study, the following information was extracted: authors, publication year, study setting, diagnostic criteria for EIPH, exercise testing methods, sample size, and the mean values and standard deviations of cardiac function indicators (CO, LVEF, mPAP, PVR, E/e′, e′, E/A, LVEDD, LVESD, SV).

### 2.5 Quality Assessment

The quality of observational studies was assessed using the Newcastle–Ottawa scale (NOS) [27]. Studies scoring ≥7 stars were considered of good quality, 5–6 stars as moderate quality, and 4 stars or below as poor quality.

### 2.6 Statistical Analysis

We conducted a meta-analysis to compare cardiac function indices between individuals with EIPH and non-EIPH controls, using continuous variables as primary outcomes. Summary effect sizes for continuous outcomes were expressed as mean differences (MDs). When continuous outcomes were reported as medians with interquartile ranges (IQRs), means and standard deviations (SDs) were estimated using the validated methods described by Wan et al. [28], assuming approximate normality of the underlying distributions, before inclusion in the meta-analysis. The inverse-variance-weighted methods were applied to combine summary MDs using random-effects models to minimize the effect of between-study heterogeneity [29]. Statistical heterogeneity was assessed using the I^2^ statistic and Cochran’s Q test. I^2^ values of <25%, 25–50%, and >50% were interpreted as indicating low, moderate, and high heterogeneity, respectively [30].

Subgroup analyses were conducted to explore potential sources of heterogeneity. Prespecified subgroup analyses were planned to explore potential sources of heterogeneity according to: (1) underlying disease category (connective tissue disease-related, heart disease-related, and dyspnea-related), (2) diagnostic threshold (mPAP/CO slope >3.0 mmHg/L/min vs. alternative criteria), (3) assessment modality (invasive vs. non-invasive), (4) body position during exercise when measuring indicators (supine vs. semi-supine vs. upright), and (5) intensity of exercise protocol (symptom-limited vs. fixed-duration). To avoid spurious findings due to sparse data, subgroup analyses and meta-regression were not performed for outcomes with five or fewer available studies. For a given outcome, a subgroup comparison was conducted only when at least two subgroups contained two or more studies. Subgroups comprising a single study were excluded from between-subgroup comparisons. If excluding single study subgroups left only one eligible subgroup, no subgroup comparison was performed.

Meta-regression was conducted to explore the influence of study-level covariates on effect estimates, with the proportion of female participants and age included as independent variables. Sensitivity analyses were conducted using a leave-one-out approach, sequentially excluding each study, and additionally restricted to higher-quality studies (NOS ≥7) to evaluate the influence of individual studies and study quality on the pooled estimates.

Publication bias was evaluated through funnel plot asymmetry tests and independent Egger’s linear regression analyses [31]. When significant bias was identified (*p* < 0.05), the trim-and-fill method was applied to estimate the number of potentially missing studies and adjust effect sizes accordingly. All statistical analyses were two-sided, and *p* < 0.05 was considered significant. Comprehensive Meta-Analysis (CMA) version 3 (Biostat, Inc., Englewood, NJ, USA) and R version 4.4.3 (R Foundation for Statistical Computing, Vienna, Austria) were used for all statistical analyses.

## 3. Results

### 3.1 Overview of Obtained Literature

The systematic search yielded 7718 records. After removing duplicates and screening titles and abstracts, 71 studies were selected for full-text review. Of these, 17 met the predefined inclusion criteria and were included in the final analysis. Additionally, one relevant study was identified through reference list screening, resulting in the inclusion of 18 studies (Fig. 1).

**Fig. 1.**
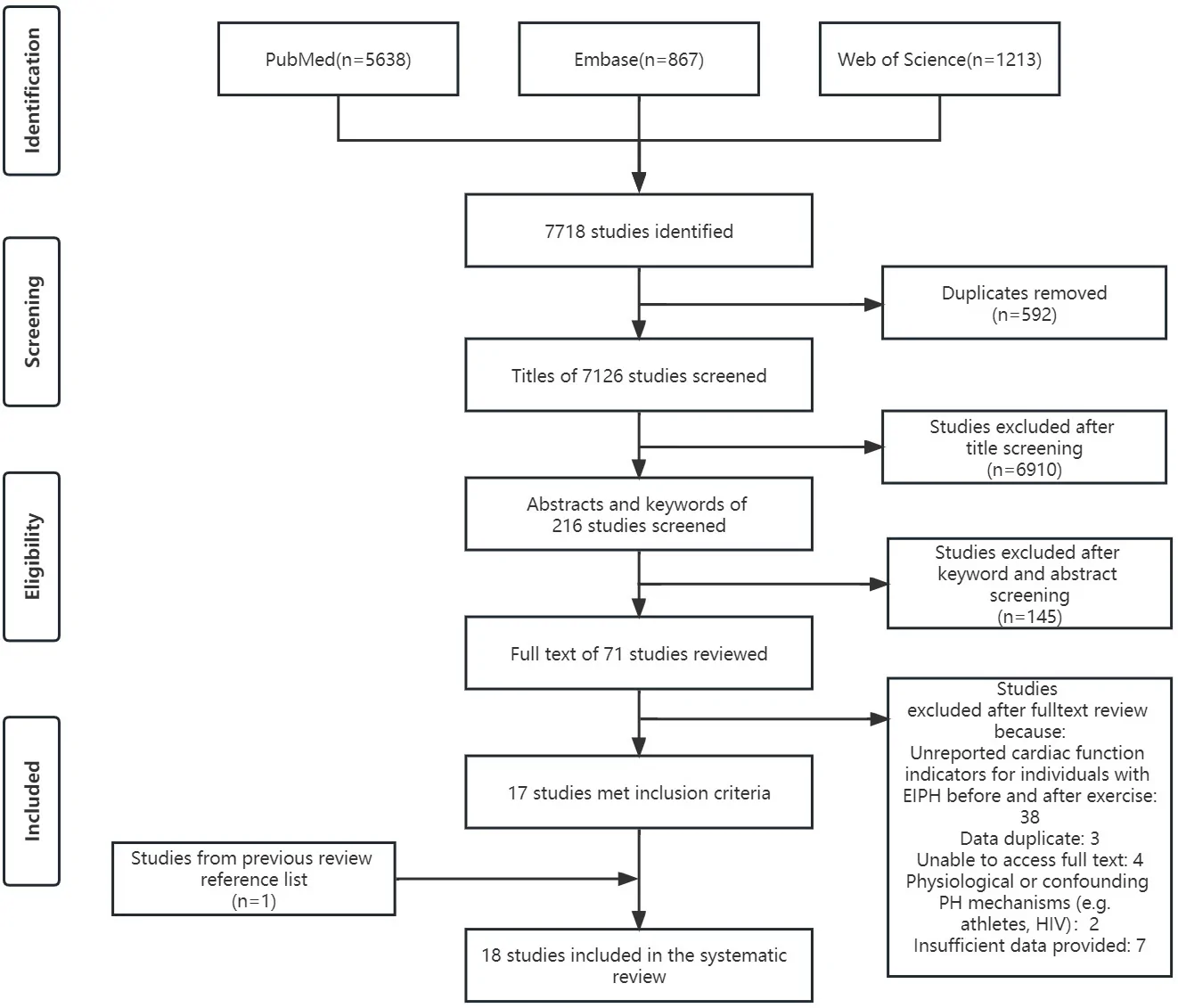
**Preferred reporting items for systematic reviews and meta-analyses (PRISMA) flowchart**. EIPH, Exercise-induced pulmonary hypertension.

The 10 cardiac function indicators reported in the 18 studies were pulmonary hemodynamic parameters (mPAP, PVR, and CO); LV diastolic function (e′ wave velocity, E/e′, and E/A); and LV systolic function (LVEF, LVEDD, LVESD, and SV). Table 1 (Ref. [1,24,32,33,34,35,36,37,38,39,40,41,42,43,44,45,46,47]) summarizes the main characteristics of the review and the individual studies. Of the 18 included studies, 8 (44.4%) were cross-sectional and 10 (55.6%) were cohort studies. Study participants included patients with systemic sclerosis, connective tissue disease, chronic dyspnea on exertion, preserved LVEF, mitral regurgitation, aortic stenosis (AS), hypertrophic cardiomyopathy, chronic obstructive pulmonary disease (COPD), and primary or secondary mitral regurgitation, among others. EIPH was defined using various invasive and non-invasive criteria, most commonly mPAP >30 mmHg, pulmonary artery systolic pressure (PASP) ≥50 mmHg, systolic pulmonary arterial pressure (sPAP) >50–60 mmHg, ΔmPAP/ΔCO slope >3, or tricuspid regurgitation peak gradient (TRPG) ≥50 mmHg during exercise. Diagnostic instruments included right-heart catheterization, Doppler echocardiography, and pulmonary gas-exchange analysis. Cardiac function was assessed primarily through exercise testing methods such as supine or semi-supine bicycle ergometry, symptom-limited graded cycle ergometry, Master’s two-step test, and upright ramp protocol cardiopulmonary exercise testing (CPET), with continuous monitoring of echocardiographic parameters, minute ventilation, or invasive hemodynamic parameters.

**Table 1.** **Characteristics of the included studies**.

Author	Sample size		Country	Population	EIPH criteria	Hemodynamic assessment	Study design	NOS score
	Non-EIPH	EIPH						
Miyanaga et al. (2019) [35]	17	7	Japan	CTD	mPAP >30 mmHg and TPR >3 WU at exercise	Exercise RHC	Cross-sectional	6
Suzuki et al. (2015) [37]	361	133	Japan	SS	post-exercise systolic PAP ≥50 mmHg	Post-exercise echocardiography	Cohort	7
Saggar et al. (2010) [36]	15	21	US	SS	mPAP >30 mmHg; PCWP ≤18 mmHg; TPG ≥15 mmHg	Exercise RHC	Cross-sectional	7
Kanazawa et al. (2024) [32]	21	19	Japan	SS	mPAP/CO slope >3	Exercise RHC	Cross-sectional	6
Voilliot et al. (2014) [39]	24	21	Belgium	SS	sPAP >50 mmHg at exercise	Exercise echocardiography	Cross-sectional	6
Tolle et al. (2008) [38]	16	78	US	Apparently healthy	Resting mPAP <25 mmHg and exercise mPAP ≥30 mmHg with PCWP <20 mmHg and PVR ≥80 dyn·s·cm^–5^	Invasive CPET	Cross-sectional	7
Ho et al. (2020) [1]	418	296	US	Dyspnea on exertion	PAP/CO slope >3 mmHg/L/min	Exercise RHC	Cohort	6
Lim et al. (2017) [24]	947	436	Korea	Exertional dyspnea or chest discomfort with preserved LVEF	PASP ≥50 mmHg	Post-exercise echocardiography	Cross-sectional	5
Ha et al. (2009) [43]	261	135	Korea	Exertional dyspnea or chest discomfort with preserved LVEF	PASP ≥50 mmHg at 50 W of exercise	Exercise echocardiography	Cross-sectional	7
Shim et al. (2011) [41]	327	171	Korea	Apparently healthy	PASP ≥50 mmHg at 50 W of exercise	Exercise echocardiography	Cohort	8
Amano et al. (2022) [42]	66	57	Japan	MR	sPAP ≥60 mmHg or TRPG ≥50 mmHg during exercise	Exercise echocardiography	Cohort	5
Magne et al. (2011) [34]	44	45	Belgium	MR	sPAP >60 mmHg	Exercise echocardiography	Cohort	7
Magne et al. (2015) [46]	43	59	Belgium	MR	sPAP >60 mmHg at exercise	Exercise echocardiography	Cohort	6
Lancellotti et al. (2015) [45]	96	63	Belgium	MR	sPAP >60 mmHg	Exercise echocardiography	Cohort	7
Lancellotti et al. (2012) [44]	47	58	Belgium	AS	sPAP >60 mmHg	Exercise echocardiography	Cohort	6
Kusunose et al. (2019) [33]	186	57	Japan	SS or CTD	ΔmPAP/ΔCO >3.3 and/or exercise mPAP >25 mmHg	Exercise echocardiography	Cohort	6
Re et al. (2022) [47]	145	37	Italy	HCM	sPAP >40 mmHg during exercise	Exercise echocardiography	Cohort	8
Skjørten et al. (2018) [40]	29	42	Norway	COPD	Resting mPAP <25 with ΔmPAP/ΔCO slope >3	Exercise RHC	Cross-sectional	6

Abbreviations: EIPH, exercise-induced pulmonary hypertension; CTD, connective tissue disease; SS, systemic sclerosis; MR, mitral regurgitation; LVEF, left ventricular ejection fraction; AS, aortic stenosis; COPD, chronic obstructive pulmonary disease; mPAP, mean pulmonary arterial pressure; sPAP, systolic pulmonary arterial pressure; HCM, hypertrophic cardiomyopathy; CO, cardiac output; WU, wood units; PVR, pulmonary vascular resistance; PASP, pulmonary artery systolic pressure; RHC, right heart catheterization; CPET, cardiopulmonary exercise testing; NOS, Newcastle–Ottawa scale; TRPG, tricuspid regurgitation peak gradient; TPG, transpulmonary pressure gradient; PCWP, pulmonary capillary wedge pressure; TPR, total pulmonary resistance.

### 3.2 Pulmonary Hemodynamic Parameters

A total of 12 records [1,24,32,33,34,35,36,37,38,39,40,41] reported associations between EIPH and pulmonary hemodynamic parameters. Eight studies [1,32,33,35,36,38,39,40] showed significantly higher mPAP values among individuals with EIPH than among non-EIPH subjects, both at rest (MD: 3.70 mmHg; 95% confidence interval (CI): 2.24 to 5.17; *p* < 0.001) and under exercise condition (MD: 10.23 mmHg; 95% CI: 8.60 to 11.85; *p* < 0.001) (Fig. 2A). A total of 11 studies [1,24,32,33,34,35,36,37,39,40,41] found that, compared with the non-EIPH participants, individuals with EIPH had significantly lower CO under the exercise condition (MD: –1.50 L/min; 95% CI: –2.45 to –0.54; *p* < 0.001), and tended to have lower CO at rest (MD: –0.23 L/min; 95% CI: –0.47 to 0.01; *p* = 0.06) (Fig. 2B). Differences in PVR values between EIPH and non-EIPH subjects were reported in eight studies [32,35,36,37,38,39,40,41], indicating that individuals with EIPH had a higher PVR at rest (MD: 0.51 WU; 95% CI: 0.33 to 0.69; *p* < 0.001) and under exercise conditions (MD: 1.30 WU; 95% CI: 0.82 to 1.78; *p* < 0.001) compared with those without EIPH (Fig. 2C).

**Fig. 2.**
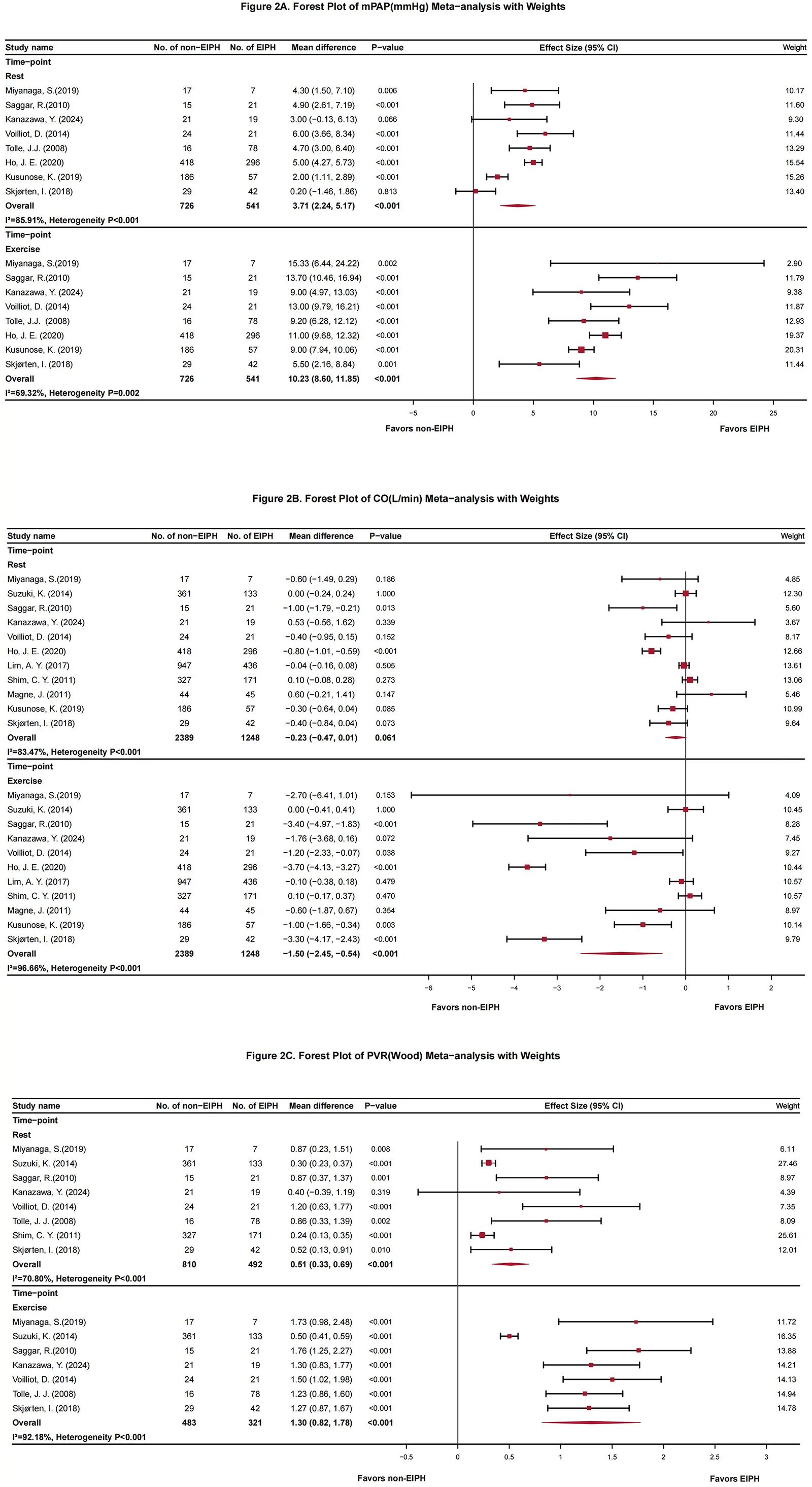
**Forest plot comparison of the differences in pulmonary hemodynamic parameters between exercise-induced pulmonary hypertension (EIPH) and non-EIPH groups at rest and under exercise conditions**. (A) Forest Plot of mPAP (mmHg) Meta-analysis with Weights; (B) Forest Plot of cO (L/min) Meta-analysis with Weights; (C) Forest Plot of PVR (Wood) Meta-analysis with Weights.

### 3.3 LV Diastolic Function

In the 15 analytical studies [24,32,33,34,35,37,39,40,41,42,43,44,45,46,47] evaluating the associations between EIPH and LV diastolic functions, 10 studies [24,32,34,37,39,41,42,43,44,45] reported the effect of EIPH on e′ wave velocity. Participants with EIPH had a lower e′ wave velocity than those without EIPH both at rest (MD: –0.99 cm/s; 95% CI: –1.49 to –0.50; *p* < 0.001) and under exercise conditions (MD: –0.86 cm/s; 95% CI: –1.55 to –0.17; *p* = 0.01) (Fig. 3A). Fourteen studies [24,32,33,34,35,37,39,40,41,43,44,45,46,47] reported that the MDs of E/e′ ratio in the EIPH group were higher than in non-EIPH group by 1.48 (95% CI: 0.80 to 2.16; *p* < 0.001) at rest and 2.46 (95% CI: 1.57 to 3.34; *p* < 0.001) under exercise conditions (Fig. 3B). No significant differences in E/A were found between EIPH and non-EIPH individuals, either at rest or under exercise conditions (Fig. 3C).

**Fig. 3.**
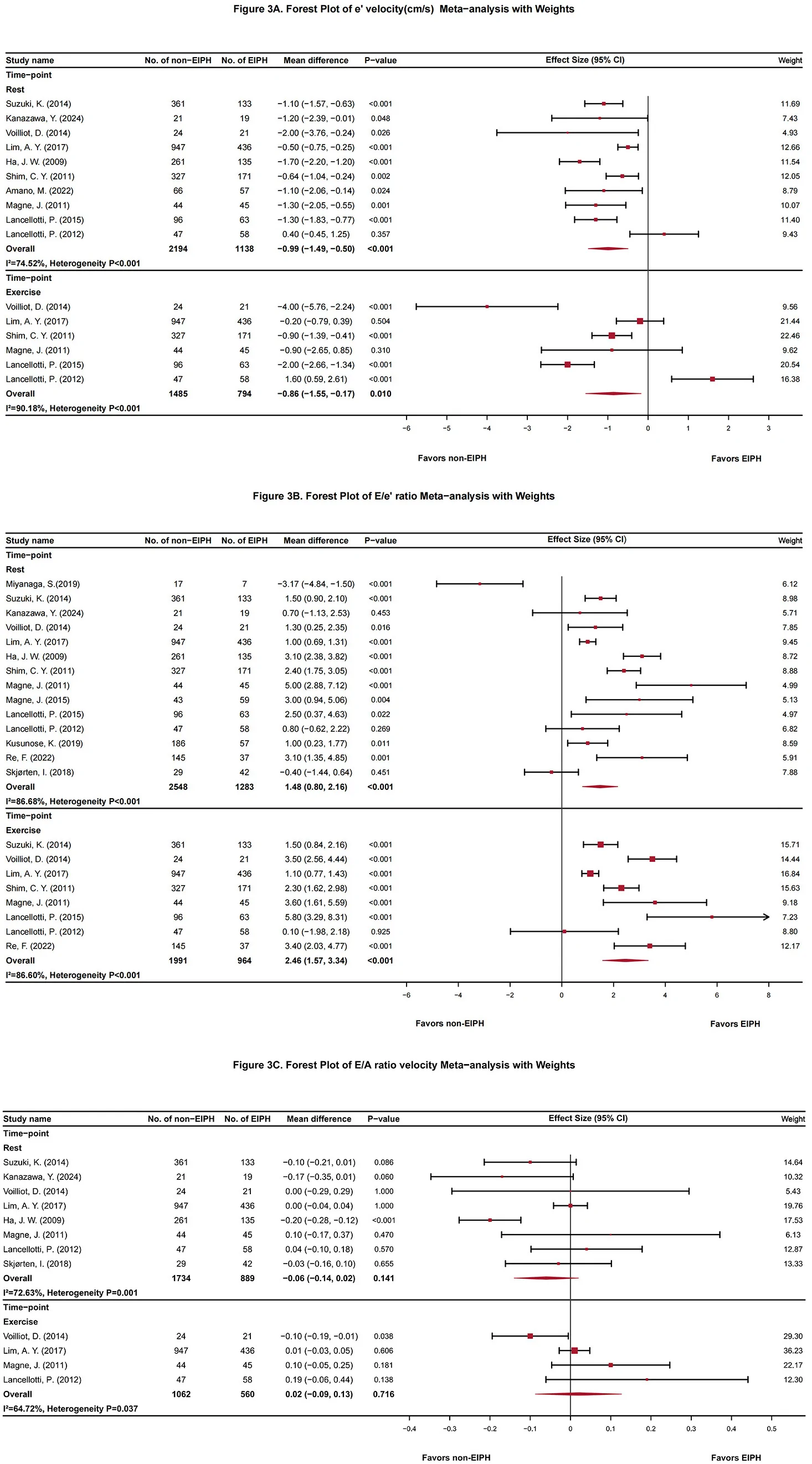
**Forest plot of the differences in left ventricular (LV) diastolic function indicators between EIPH and non-EIPH groups at rest and under exercise conditions**. (A) Forest Plot of e' velocity (cm/s) Meta-analysis with Weights; (B) Forest Plot of E/e' ratio Meta-analysis with Weights; (C) Forest Plot of E/A ratio velocity Meta-analysis with Weights.

### 3.4 LV Systolic Function

Sixteen studies [1,24,32,33,34,35,37,39,40,41,42,43,44,45,46,47] reported associations between EIPH and LV systolic function. In the pooled analysis of 6 studies [1,34,39,44,45,46] revealed that individuals with EIPH had a significantly lower LVEF under exercise conditions (MD: –1.96; 95% CI: –3.25 to –0.67; *p* = 0.003), whereas no significant differences were found at rest (Fig. 4A). Six studies [24,37,41,42,43,46] showed a significantly lower LVESD (MD: –0.54 mm; 95% CI: –0.94 to –0.15; *p* = 0.007) in individuals with EIPH compared with non-EIPH participants (Fig. 4B). No significant differences between EIPH and non-EIPH individuals were found for LVEDD or SV, either at rest or under exercise conditions (Fig. 4B,C).

**Fig. 4.**
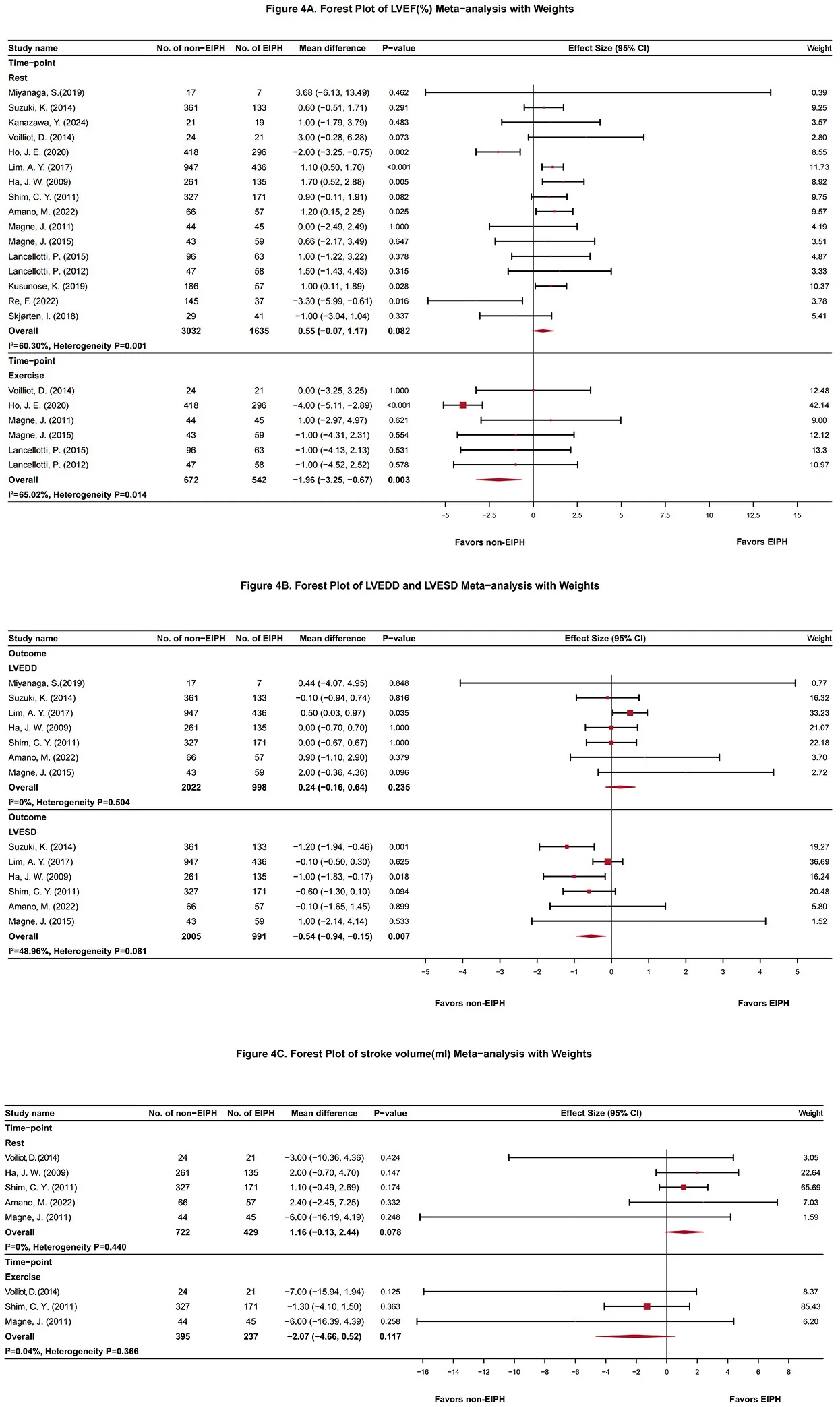
**Forest plot of the differences in LV systolic function indicators between EIPH and non-EIPH groups at rest and under exercise conditions**. (A) Forest Plot of LVEF (%) Meta-analysis with weights; (B) Forest Plot of LVEDD andLVESD Meta-analysis with Weights; (C) Forest Plot of stroke volume (mL) Meta-analysis with Weights.

### 3.5 Subgroup Analysis, Sensitivity Analyses, and Meta-Regression Analysis

Significant subgroup differences were observed according to assessment modality (invasive vs. non-invasive) for CO at rest and during exercise, and for LVEF and E/e′ at rest (all *p* for subgroup difference <0.01). Meta-regression analyses indicated that assessment modality accounted for a substantial proportion of between-study heterogeneity for CO (R^2^ = 0.81 at rest and R^2^ = 0.96 during exercise). A statistically significant subgroup difference was also observed for PVR at rest (*p* for subgroup difference = 0.02). However, substantial heterogeneity was present within subgroups, particularly among non-invasive studies, indicating that this finding should be interpreted with caution.

When stratified by the EIPH definition, significant subgroup differences were observed for CO at rest and during exercise, whereas no significant subgroup differences were observed for other cardiac function indicators. Exploratory subgroup analyses stratified by underlying disease category demonstrated generally consistent effect directions across populations; however, for most outcomes, no statistically significant differences between groups were observed. Meanwhile, when stratified by participant characteristics, dyspnea and connective tissue disease- or CVD-related conditions were not identified as potential sources of heterogeneity. Nonetheless, E/e′ ratios at rest were significantly elevated among individuals with CVD.

Overall, body position during exercise and exercise intensity did not clearly explain the observed between-study heterogeneity for mPAP, CO, and E/e′ in the subgroup analyses. For the pooled estimate of LVEF during exercise, excluding the large-weight upright exercise position study rendered the pooled estimate from the remaining five semi-supine studies non-significant [1]. Although the direction of effect remained broadly consistent, these results indicate that the pooled finding is highly sensitive to this single study and does not allow a definitive conclusion regarding the association between EIPH and LVEF during exercise.

For PVR during exercise stratified by intensity, excluding the single fixed-duration study eliminated heterogeneity (I^2^ = 0%), indicating that this study was the primary contributor to between-study variation [37]. Importantly, the pooled estimate remained statistically significant after this exclusion, suggesting that the main finding is robust despite differences in exercise protocols.

Meta-regression analyses were conducted to examine whether age and percentage of female participants modified the pooled MDs in cardiac function indicators between individuals with EIPH and non-EIPH controls. Increasing age was significantly associated with a smaller pooled MD in mPAP between the EIPH and non-EIPH groups at both rest and during exercise, with R^2^ values of 0.54 and 1.0, respectively. Modest study-level associations were also observed between age and LVEDD and LVESD at rest. No significant associations were observed between age and other hemodynamic or functional cardiac indices.

The proportion of female participants was not significantly associated with pooled MDs for most hemodynamic and cardiac function indices, including mPAP, PVR, CO, and LVEF, at rest or during exercise. Although a higher proportion of female participants was associated with smaller pooled MDs for E/e′ and LVESD at rest, these findings were based on limited data and unstable estimates of heterogeneity; therefore, these estimates should be interpreted with caution. Detailed results of the subgroup and meta-regression analyses are presented in **Supplementary Tables 2–4** and **Supplementary Figs. 1–6**.

Other leave-one-out sensitivity analyses showed that exclusion of any single study did not substantially alter the pooled estimates. No significant differences were observed at rest or during exercise in any cardiac function indicator, indicating that the overall findings were robust (**Supplementary Table 5**).

To assess the influence of study quality on the pooled estimates, sensitivity analyses were performed that included only studies with NOS scores ≥7. For most outcomes (*e*.*g*., mPAP, PVR, E/e′, and SV at rest and during exercise; CO, e′, LVEF, LVEDD, and LVESD at rest; E/A during exercise), pooled estimates from higher-quality studies (NOS ≥7) were generally consistent in direction and significance with the main analysis, indicating that the findings were robust to exclusion of lower-quality studies. For a few outcomes (CO, e′, and LVEF during exercise; E/A during rest), the pooled estimates among NOS ≥7 studies remained in the same direction as the main analysis, but statistical significance differed, suggesting that these results may be sensitive to the inclusion of moderate-quality (NOS = 5 or 6) studies (**Supplementary Table 6**).

### 3.6 Publication Bias

Evidence of funnel plot asymmetry was observed for selected outcomes, including PVR (rest and exercise), LVEF (exercise), and E/e′ (exercise), whereas SV showed no indication of small-study effects (**Supplementary Figs. 7–24**). Trim-and-fill analyses suggested modest changes in pooled estimates due to potential small-study effects. PVR (rest and exercise), and E/e′ (exercise) remained statistically significant after adjustment, whereas LVEF during exercise became non-significant, indicating that small-study effects may have influenced this outcome.

## 4. Discussion

To our knowledge, this is the first systematic review and meta-analysis to investigate the cardiac functional profile of individuals with EIPH. Based on data from the included studies, our results provide robust evidence that EIPH is both a transient hemodynamic phenomenon and consistently associated with impaired cardiac function. This impairment is primarily characterized by early-stage diastolic dysfunction, as reflected by altered e′ and elevated E/e′ ratio. Furthermore, blunted CO and elevated mPAP and PVR suggest compromised ventricular pump function. These hemodynamic changes and chronic pressure overload trigger a harmful remodeling process in which the right ventricle thickens and dilates, eventually leading to progressive ventricular dysfunction and biventricular failure.

During exercise, elevated pulmonary pressure imposes acute strain on the right ventricle, which could trigger a ventricular septal shift toward the left ventricle and ultimately lead to biventricular diastolic dysfunction [48]. Moreover, this “ventricular interference” leads to exertional dyspnea and progressive exercise intolerance, even in the absence of resting cardiopulmonary pathology. These findings highlight that EIPH is not an independent vascular event but a systemic cardiovascular stressor that is an indicator of early hemodynamic abnormalities and a long-term risk factor for overt heart failure.

### 4.1 Association of EIPH With Pulmonary Hemodynamic Parameters

The adverse association of EIPH with pulmonary hemodynamic parameters is primarily characterized by reduced CO under exercise conditions. Notably, healthy individuals exhibit a robust elevation in CO to meet metabolic demands, whereas patients with EIPH experience an abnormal rise in mPAP and PVR that imposes significant stress on the right ventricle. Indeed, the present study found that the CO of patients with EIPH decreased by 1.50 L/min during exercise compared with healthy individuals, whereas resting differences were marginal. This suggests mild impairment of pulmonary hemodynamic parameters in EIPH, potentially triggered by a hemodynamic triad during exercise.

Prolonged exposure to high pressures may be associated with progression to resting PH and overt right heart failure (RHF) [49]. A prospective study of 90 patients with PH from Johns Hopkins Hospital and affiliated centers found that RHF was the leading cause of admission (56%) [50].

Hemodynamic data obtained at the time of PAH diagnosis revealed that mPAP and PVR were 49 mmHg and 10.8 WU, respectively, with an in-hospital mortality rate of 14% among patients with RHF. Another retrospective study demonstrated that, among patients with heart failure, each 1 mmHg increase in mPAP was associated with a 2% higher risk of all-cause mortality or heart failure hospitalization, whereas each 1 WU increase in PVR elevates this risk by 7% [51]. Furthermore, an abnormal decline in CO during exercise indicates exhaustion of RV afterload-compensatory mechanisms, which could ultimately lead to peripheral vasoconstriction and severe end-organ damage. This pattern is a critical determinant of cardiogenic shock, a condition associated with a 30-day mortality rate of 39.7%, posing a profound health risk to the EIPH population [52].

### 4.2 Association of EIPH With LV Diastolic Function

Our findings confirm that EIPH is significantly associated with impaired LV diastolic function, as evidenced by decreased e′ values and elevated E/e′ ratios. Notably, a more pronounced elevation of E/e′ ratios during exercise (MD: 2.46 vs. 1.48) compared with rest suggests a substantial increase in LV filling pressure in the EIPH population during exercise. Our findings further indicate that the difference in mean resting E/e′ ratios between EIPH and non-EIPH groups was more pronounced in studies involving CVD populations, suggesting relatively greater impairment of LV diastolic function in this subgroup. This phenomenon likely arises from chronic RV pressure overload, which may result in RV wall thickening, a leftward septal shift, and a restricted LV filling space.

Physiologically, these abnormalities are due to “heart-room interference”. Meanwhile, chronic RV pressure overload is associated with wall thickening that physically pushes into the LV filling space, thereby increasing filling resistance, a compensatory response to RV diastolic dysfunction [48]. Clinically, understanding that the ‘hidden’ pressure risk, specifically the PAWP/CO slope, is essential for differentiating between pre-capillary pulmonary vascular disease (PVD) and post-capillary left heart involvement is critical [53,54,55,56,57].

Recent studies have indicated that a PAWP/CO slope >2 mmHg/L/min represents a key indicator for identifying occult PAWP elevation, especially in individuals with resting PAWP of 12–15 mmHg, while an exercise PAWP ≥25 mmHg (supine position) has been established as a diagnostic threshold for HFpEF [53,58]. Early-stage PVD is characterized by a hemodynamic split during exercise: the mPAP/CO slope rises above 3.0 mmHg/L/min, while the PAWP/CO slope remains normal [54,55,56].

Cardiac injury induced by EIPH is associated with persistent elevation of the E/e′ ratio, thereby increasing heart failure risk in EIPH populations. In patients with heart failure with improved ejection fraction (HFimpEF), each 1-unit increase in the E/e′ ratio correlates with a 28% rise in the composite risk of heart failure rehospitalization and all-cause mortality [57]. Patients with E/e′ >13.80 exhibit a 4.48-fold higher risk of heart failure rehospitalization within 6 months compared with those with lower E/e′ values [59]. The continuous progression of EIPH progressively increases cardiac pumping load, driving ongoing E/e′ ratio elevation that further promotes RV hypertrophy and exacerbates LV diastolic dysfunction. This pathological remodeling process portends a poorer prognosis for cardiovascular diseases, sharing similar pathophysiological mechanisms with AS patients, where diastolic impairment is a primary risk factor for long-term mortality [60].

### 4.3 Association of EIPH With LV Systolic Function

Our analysis revealed a refined relationship between EIPH and LV systolic function. The modest reduction in exercise LVEF and the smaller LVESD are likely both associated with the same “heart-room interference” mechanism observed in diastolic dysfunction. However, the findings regarding exercise LVEF should be interpreted with caution, as exercise position appears to influence measured LVEF, with upright assessments potentially attenuating observable systolic responses. Clinically, the pooled MD of 1.96% is notably lower than the established minimum detectable difference (MDD) for echocardiographic LVEF measurement, which is typically approximately 4 percentage points [61]. This indicates that, at the individual patient level, such a minor change falls within measurement variability and lacks clinical relevance for diagnosing overt systolic dysfunction. Furthermore, sensitivity analyses indicated that the statistical significance of exercise LVEF was highly dependent on a single large-weight study, and the trim-and-fill analysis collectively suggests a convergent pattern of statistical fragility for this specific parameter. Therefore, this modest alteration should be viewed as an exploratory signal rather than a definitive indication of overt systolic failure.

The subgroup differences by assessment modality likely reflect inherent differences in hemodynamic quantification: invasive methods directly measure CO and pressures, whereas non-invasive techniques rely on Doppler-based estimates and geometric assumptions that affect flow-dependent indices, thereby contributing to between-study heterogeneity. These findings highlight the need for methodological standardization in non-invasive assessments.

The between-group difference observed in CO, according to the EIPH definition, likely reflects structural distinctions between mPAP/sPAP cutoff-based and slope-based criteria, as slope-based definitions explicitly incorporate the mPAP–CO relationship.

Overall, meta-regression analyses demonstrated that age was primarily associated with differences in mPAP. Similarly, sex distribution had minimal influence on pooled hemodynamic contrasts, with only isolated associations that were not consistently replicated across related parameters, suggesting that demographic composition is unlikely to be a major source of between-study heterogeneity in EIPH-related differences in cardiac function.

### 4.4 Implications

In susceptible individuals, EIPH is not a normal hemodynamic variation but an early marker of cardiac functional impairment. The observed reduction in exercise CO, coupled with abnormally increasing mPAP and PVR, signals an RV–pulmonary vascular uncoupling that is closely associated with the progression toward persistent PH and right heart failure. Crucially, the widening E/e′ ratio during exercise conditions suggests that EIPH is a vital window for identifying subclinical left-heart diastolic dysfunction. To translate these findings into practice, standardized, non-invasive stress imaging diagnostic protocols are needed and should be integrated into primary prevention strategies targeting early cardiac dysfunction in at-risk EIPH populations. Such tools are essential for distinguishing early-stage pulmonary vascular disease from age-related physiological changes or congestion associated with left-heart disease. Increasing clinical awareness of these early functional declines will facilitate timely intervention and potentially halt the remodeling process before irreversible cardiovascular damage occurs.

### 4.5 Limitations

This study has several limitations. First, this meta-analysis was based on aggregate data from published studies rather than individual participant data (IPD), limiting the ability to explore patient-level variation and effect modification. Second, substantial heterogeneity was observed across studies. Subgroup analyses and meta-regression suggested that this heterogeneity was largely explained by assessment modality, EIPH diagnostic criteria, body position of the measuring indicators during exercise, and exercise intensity. Some high R^2^ values (including R^2^ = 1) are likely artifacts of the small number of studies rather than reflecting true biological effects. Third, due to limited data availability, we were unable to perform analyses stratified by diagnostic criteria recommended by the ESC/ERS guidelines [3] to examine the relationship between EIPH and RV diastolic function. Lastly, sensitivity analyses restricted to higher-quality studies (NOS ≥7) indicated that most outcomes were robust to the exclusion of moderate-quality studies; however, a few indicators showed variation in statistical significance. Meanwhile, the existence of publication bias reflects greater methodological variability. Trim-and-fill analyses suggested that small-study effects may have influenced the pooled estimate of exercise LVEF; therefore, this estimate should be interpreted with caution.

## 5. Conclusions

This meta-analysis demonstrates that EIPH represents a distinct early pathological phenotype that signals impaired cardiac function. Our findings indicate that EIPH is associated with subclinical biventricular dysfunction, where adverse ventricular interdependence contributes to left-heart diastolic impairment. Consequently, EIPH serves as a critical clinical window for detecting subclinical heart failure that remains undetectable at rest and should be recognized for early risk stratification. Future research should prioritize tracking disease progression and conducting prospective trials to determine whether early intervention can prevent progression to overt cardiovascular failure, a crucial step toward improving long-term cardiovascular outcomes.

## Data Availability

Data described in the manuscript will be made available upon reasonable request to the corresponding authors.

## Abbreviations

EIPH, Exercise-induced pulmonary hypertension; mPAP, Mean pulmonary artery pressure; PVR, Pulmonary vascular resistance; CO, Cardiac output; LAP, Left atrial pressure; PAP, Pulmonary artery pressure; PH, Pulmonary hypertension; e′, Mitral annular early diastolic velocity; E/e′, Early transmitral flow velocity to early mitral annular velocity ratio; E/A, Early diastolic filling velocity to late diastolic filling velocity ratio; LVEF, Left ventricular ejection fraction; LVEDD, Left ventricular end-diastolic diameter; LVESD, Left ventricular end-systolic diameter; SV, Stroke volume; MD, Mean difference; SE, Standard error; CIs, Confidence Intervals; PASP, Pulmonary artery systolic pressure; RHF, Right heart failure; WU, Wood Units; HFimpEF, Heart failure with improved ejection fraction; AS, Aortic stenosis; iCPET, Invasive cardiopulmonary exercise test.
