# Supplementary material for: Association of Exercise-Induced Pulmonary Hypertension and Echocardiographic Parameters of Cardiac Function: A Systematic Review and Meta-Analysis
Source: Rev Cardiovasc Med. 2026 Oct 20;27(10):51027. doi: 10.31083/RCM51027 (PMC13732670; doi:10.31083/RCM51027)
Supplement: Supplementary file 1 [file 2153-8174-27-10-51027-s1.zip › Supplementary Material/Supplementary Material.docx]

**Supplementary Table 1. search strategy**

**Search terms for** **PubMed (n=5, 638), until April 30, 2025**

**Exercise-Induced Pulmonary Hypertension(n=26, 391)**

#1 Pulmonary Hypertension[MeSH] OR Pulmonary Hypertension[Title/Abstract] OR Pulmonary Arterial Hypertension[Title/Abstract] OR Pulmonary Artery Pressure[Title/Abstract] OR PAH[Title/Abstract] OR PH[Title/Abstract] OR Pulmonary Vascular[Title/Abstract]

#2 exercise[Title/Abstract] OR Exercise-Induced[Title/Abstract] OR exercise test[Title/Abstract] OR exercise tolerance[Title/Abstract] OR physical[Title/Abstract]

#3 (#1 AND #2)

**Cardiac Function/Structure(n=2, 128, 316)**

#4 ("Ventricular Function"[Mesh] OR "Ventricular Dysfunction"[Mesh] OR "Ventricular Dysfunction, Left"[Mesh] OR "Ventricular Dysfunction, Right"[Mesh] OR "Atrial Function"[Mesh] OR "Myocardial Contraction"[Mesh] OR "Coronary Circulation"[Mesh]) OR

("Heart Function Tests"[Mesh] OR "Cardiac Output"[Mesh] OR "Stroke Volume"[Mesh]) OR

("Cardiac Volume"[Mesh] OR "Echocardiography"[Mesh] OR "Fetal Heart"[Mesh] OR "Heart"[Mesh] AND ("Endocardium"[Mesh] OR "Heart Atria"[Mesh] OR "Heart Septum"[Mesh] OR "Heart Ventricles"[Mesh] OR "Myocardium"[Mesh] OR "Pericardium"[Mesh] OR "Hypertrophy, Left Ventricular"[Mesh])) OR

(((heart[Title/Abstract] OR cardiac[Title/Abstract] OR ventricular[Title/Abstract] OR ventricle*[Title/Abstract] OR biventric*[Title/Abstract] OR myocard*[Title/Abstract] OR septal[Title/Abstract] OR septum[Title/Abstract] OR systol*[Title/Abstract] OR diastol*[Title/Abstract] OR endsystol*[Title/Abstract] OR enddiastol*[Title/Abstract] OR hyperdynam*[Title/Abstract]) AND (function*[Title/Abstract] OR d?sfunct*[Title/Abstract] OR malfunct*[Title/Abstract] OR contract*[Title/Abstract] OR relaxat*[Title/Abstract] OR performanc*[Title/Abstract] OR efficienc*[Title/Abstract] OR mechanoenerg*[Title/Abstract] OR mechano-energ*[Title/Abstract] OR programming[Title/Abstract] OR (TDV[Title/Abstract] NOT vortex*[Title/Abstract]))) OR ((systol*[Title/Abstract] OR diastol*[Title/Abstract] OR ventricular[Title/Abstract] OR LV[Title/Abstract]) AND impair*[Title/Abstract]) OR "tissue doppler"[Title/Abstract] OR MPI[Title/Abstract] OR Tei-ind*[Title/Abstract] OR MEEi[Title/Abstract])) OR

((card*[Title/Abstract] OR ventric*[Title/Abstract] OR myocard*[Title/Abstract] OR concentric[Title/Abstract]) AND remodel*[Title/Abstract]) OR

((cardiac output[Title/Abstract] OR cardiac index[Title/Abstract] OR (pump function*[Title/Abstract]) OR ((stroke volume*[Title/Abstract] OR enddiastol*[Title/Abstract] OR end-diastol*[Title/Abstract] OR endsystol*[Title/Abstract] OR end systol*[Title/Abstract] OR late diastol*[Title/Abstract]) AND volume*[Title/Abstract]) OR ejection fraction*[Title/Abstract] OR VEF[Title/Abstract] OR LVEF*[Title/Abstract] OR RVEF[Title/Abstract] OR ((ventric* output[Title/Abstract] OR ventric* volume*[Title/Abstract] OR ventric* ejection*[Title/Abstract] OR ventric* enddiastol*[Title/Abstract] OR ventric* end-diast*[Title/Abstract] OR ventric* late-diastol*[Title/Abstract] OR ventric* endsystol*[Title/Abstract] OR ventric* end-systol*[Title/Abstract])))) OR

(d?synchron*[Title/Abstract] OR DYS[Title/Abstract] OR 2CDYS[Title/Abstract] OR 1CDYS[Title/Abstract])) OR

(((global[Title/Abstract] OR segment*[Title/Abstract] OR longitudin*[Title/Abstract] OR systolic[Title/Abstract] OR diastolic[Title/Abstract] OR peak*[Title/Abstract] OR rate[Title/Abstract] OR imag*[Title/Abstract] OR indices[Title/Abstract] OR index[Title/Abstract]) AND (strain[Title/Abstract] OR motion*[Title/Abstract] OR deformat*[Title/Abstract])) OR GSR[Title/Abstract] OR "frame rate*"[Title/Abstract])) OR

(echocardiogra*[Title/Abstract] OR echo-cardiogra*[Title/Abstract] OR ECG[Title/Abstract] OR fECG[Title/Abstract] OR EFE[Title/Abstract] OR ((echo*[Title/Abstract] OR imaging[Title/Abstract] OR MRI[Title/Abstract]) AND (cardiac[Title/Abstract] OR heart*[Title/Abstract]))) OR

(speckle*[Title/Abstract])) OR

((E-E[Title/Abstract] OR E-A[Title/Abstract]) AND (systol*[Title/Abstract] OR diastol*[Title/Abstract] OR veloc*[Title/Abstract])) OR (((E-E OR E-A) AND ratio*[Title/Abstract]) OR ((diastole[Title/Abstract] OR diastolic[Title/Abstract] OR systole[Title/Abstract] OR systolic[Title/Abstract] OR veloc*[Title/Abstract] OR decelerat*[Title/Abstract]) AND "E"[Title/Abstract]))) OR

(((mitral[Title/Abstract] OR tricuspid*[Title/Abstract] OR septal[Title/Abstract] OR lateral[Title/Abstract]) AND ("E"[Title/Abstract] OR wave*[Title/Abstract] OR veloc*[Title/Abstract] OR inflow[Title/Abstract] OR systol*[Title/Abstract] OR diastol*[Title/Abstract])) OR MAPSE[Title/Abstract] OR TAPSE[Title/Abstract] OR E-vel[Title/Abstract])) OR

(velocity time integral*[Title/Abstract] OR VTI[Title/Abstract] OR (valv*[Title/Abstract] AND veloc*[Title/Abstract]))) OR

((systol*[Title/Abstract] AND excurs*[Title/Abstract]) OR ((annulus[Title/Abstract] OR annular[Title/Abstract]) AND (diamet*[Title/Abstract] OR plain[Title/Abstract]))) OR

(cardiac[Title/Abstract] AND (structure*[Title/Abstract] OR shape*[Title/Abstract] OR diamet*[Title/Abstract] OR axis[Title/Abstract] OR size*[Title/Abstract] OR radius[Title/Abstract] OR chang*[Title/Abstract] OR alterat*[Title/Abstract] OR biometr*[Title/Abstract] OR developm*[Title/Abstract] OR workload[Title/Abstract]))) OR

(heart[Title/Abstract] AND (structure*[Title/Abstract] OR shape*[Title/Abstract] OR diamet*[Title/Abstract] OR chang*[Title/Abstract] OR alterat*[Title/Abstract] OR biometr*[Title/Abstract] OR developm*[Title/Abstract] OR workload[Title/Abstract]))) OR

(((heart[Title/Abstract] OR card*[Title/Abstract] OR left[Title/Abstract] OR right[Title/Abstract]) AND ventric*[Title/Abstract]) OR (LV[Title/Abstract] NOT leg-volum*[Title/Abstract]) OR (RV[Title/Abstract] NOT residual volum*[Title/Abstract]) OR LVOT[Title/Abstract] OR RVOT[Title/Abstract] OR outflow-tract*[Title/Abstract])) OR

(atrium[Title/Abstract] OR atria[Title/Abstract] OR interatr*[Title/Abstract] OR inter-atr*[Title/Abstract])) OR

(((LA[Title/Abstract] OR left atri*[Title/Abstract]) AND atria*[Title/Abstract]) OR lA-Ao*[Title/Abstract])) OR

(myocardium[Title/Abstract])) OR

((heart[Title/Abstract] OR cardiac[Title/Abstract] OR ventr*[Title/Abstract] OR interventr*[Title/Abstract] OR atrial[Title/Abstract]) AND sept*[Title/Abstract])) OR

((f?etal[Title/Abstract] OR f?etus*[Title/Abstract]) AND (myocard*[Title/Abstract] OR iVS[Title/Abstract])) OR

((aort*[Title/Abstract] AND (root*[Title/Abstract] OR central[Title/Abstract] OR ascend*[Title/Abstract] OR ejection*[Title/Abstract] OR strain[Title/Abstract] OR augmentat*[Title/Abstract] OR AIx[Title/Abstract])) OR AoV[Title/Abstract])) OR

(epicardium[Title/Abstract] OR epicardial[Title/Abstract] OR pericardium[Title/Abstract] OR pericardial[Title/Abstract] OR (cardiac[Title/Abstract] AND fat[Title/Abstract]) OR EFT[Title/Abstract] OR hypo-echogen*[Title/Abstract] OR hypoechogen*[Title/Abstract])) OR

((ventric*[Title/Abstract] AND (wall*[Title/Abstract] OR cavit*[Title/Abstract])) OR PWLV*[Title/Abstract] OR circumferential fib*[Title/Abstract] OR mVcf[Title/Abstract] OR VcF[Title/Abstract])) OR

((((intraventric*[Title/Abstract] OR ventric*[Title/Abstract] OR biventr*[Title/Abstract]) NOT ((intraventric*[Title/Abstract] OR ventric*[Title/Abstract]) AND (bleed*[Title/Abstract] OR blood-loss*[Title/Abstract] OR h?emorr*[Title/Abstract]))) OR septal[Title/Abstract] OR septum[Title/Abstract] OR IVS[Title/Abstract] OR myocard*[Title/Abstract] OR atrium[Title/Abstract] OR atria*[Title/Abstract]) AND (hypertroph*[Title/Abstract] OR thick*[Title/Abstract] OR width*[Title/Abstract] OR strain*[Title/Abstract] OR mass[Title/Abstract] OR masses[Title/Abstract] OR wall*[Title/Abstract] OR geometr*[Title/Abstract] OR circumfer*[Title/Abstract] OR diamet*[Title/Abstract] OR distanc*[Title/Abstract] OR dimension*[Title/Abstract] OR area[Title/Abstract] OR volum*[Title/Abstract] OR globul*[Title/Abstract] OR global[Title/Abstract] OR segment*[Title/Abstract] OR spher*[Title/Abstract] OR architectur*[Title/Abstract] OR structure*[Title/Abstract] OR shape*[Title/Abstract] OR axis[Title/Abstract] OR size*[Title/Abstract] OR radius[Title/Abstract] OR chang*[Title/Abstract] OR biometr*[Title/Abstract] OR developm*[Title/Abstract] OR workload[Title/Abstract] OR deformat*[Title/Abstract] OR lengthen*[Title/Abstract] OR morphometr*[Title/Abstract] OR morphofunct*[Title/Abstract] OR velocit*[Title/Abstract] OR motion*[Title/Abstract] OR TDI[Title/Abstract] OR ratio[Title/Abstract] OR ratios[Title/Abstract] OR index[Title/Abstract] OR indices[Title/Abstract]) OR ((cardiac[Title/Abstract] OR eccentr*[Title/Abstract]) AND hypertroph*[Title/Abstract]) OR LVMI[Title/Abstract] OR LVM[Title/Abstract] OR LVSI[Title/Abstract] OR spheri* ind*[Title/Abstract] OR TDI-velocit*[Title/Abstract] OR EDIVS[Title/Abstract] OR EDLVPW[Title/Abstract] OR relative wall thickness[Title/Abstract] OR RWT[Title/Abstract] OR LVPWd[Title/Abstract] OR LVID[Title/Abstract] OR LIVDs[Title/Abstract] OR LIVDd[Title/Abstract] OR IVSd[Title/Abstract])) OR

((fraction*[Title/Abstract] OR midwall[Title/Abstract] OR ventric*[Title/Abstract]) AND shorten*[Title/Abstract])

**Cardiovascular Risk(n=3, 192, 305)**

#5 "cardiovascular diseases"[MeSH] OR "coronary artery disease"[MeSH] OR "atherosclerosis"[ MeSH] OR "coronary disease"[MeSH] OR "myocardial infarction"[MeSH] OR "myocardial ischemia"[MeSH] OR "stroke"[MeSH] OR "cardiovascular disease" [All Fields] OR "coronary artery disease" [All Fields] OR "atherosclerosis" [All Fields] OR "coronary disease"[All Fields] OR "myocardial infarction"[All Fields] OR "myocardial ischemia"[All Fields] OR "stroke"[All Fields]

#6 "humans"[MeSH Terms]

**((#3 AND #4 OR (#3 AND #5)) AND #6(n=5, 638)**

**Supplementary Table 2. Subgroup analyses**

| subgroup | No. of available study | Heterogeneity | | Pooled Effect Size | | p for group different | R2 |
| --- | --- | --- | --- | --- | --- | --- | --- |
|  |  | P-Value | I² | MD (95% CI) | P-Value |  |  |
| **Hemodynamic Assessment** | | | | | | | |
| **mPAP(rest)** |  |  |  |  |  | **0.98** | **0.00** |
| Invasive | 6 | <0.01 | 82.24 | 3.70 (1.88, 5.52) | <0.001 |  |  |
| Non-invasive | 2 | <0.01 | 89.78 | 3.75 (0.71, 6.79) | 0.016 |  |  |
| **mPAP(exercise)** |  |  |  |  |  | 0.81 | 0.00 |
| Invasive | 6 | 0.01 | 67.39 | 10.12 (7.79, 12.44) | <0.001 |  |  |
| Non-invasive | 2 | 0.02 | 81.37 | 10.64 (7.12, 14.15) | <0.01 |  |  |
| **CO(Rest)** |  |  |  |  |  | <0.01 | 0.81 |
| Invasive | 5 | 0.09 | 51.02 | -0.64 (-0.89, -0.38) | <0.001 |  |  |
| Non-invasive | 6 | 0.13 | 41.53 | -0.04 (-0.20, 0.13) | 0.670 |  |  |
| **CO(Exercise)** |  |  |  |  |  | <0.01 | 0.96 |
| Invasive | 5 | 0.36 | 8.08 | -3.41 (-3.94, -2.89) | <0.01 |  |  |
| Non-invasive | 6 | 0.02 | 63.14 | -0.25 (-0.57, 0.08) | 0.130 |  |  |
| **PVR(rest)** |  |  |  |  |  | 0.02 | 0.54 |
| Invasive | 5 | 0.66 | 0.00 | 071 (0.45, 0.97) | <0.01 |  |  |
| Non-invasive | 3 | <0.01 | 81.21 | 0.34 (0.17, 0.51) | <0.01 |  |  |
| **PVR(exercise)** |  |  |  |  |  | 0.16 | 0.59 |
| Invasive | 5 | 0.40 | 0.84 | 1.43 (1.03, 1.83) | <0.01 |  |  |
| Non-invasive | 2 | <0.01 | 93.91 | 0.92 (0.34, 1.50) | 0.002 |  |  |
| **LVEF(rest)** |  |  |  |  |  | 0.001 | 0.76 |
| Invasive | 4 | 0.18 | 38.41 | -1.20 (-2.34, -0.07) | 0.040 |  |  |
| Non-invasive | 12 | 0.22 | 22.49 | 0.95 (0.48, 1.42) | <0.001 |  |  |
| **E/e’(rest)** |  |  |  |  |  | <0.01 | 0.32 |
| Invasive | 3 | <0.01 | 81.64 | -0.93 (-2.29, 0.43) | 0.180 |  |  |
| Non-invasive | 11 | <0.01 | 81.96 | 2.03 (1.38, 2.69) | <0.001 |  |  |
| **E/A(rest)** |  |  |  |  |  | 0.67 | 0.00 |
| Invasive | 2 | 0.21 | 35.46 | -0.09 (-0.27, 0.08) | 0.300 |  |  |
| Non-invasive | 6 | <0.01 | 78.81 | -0.05 (-0.15, 0.05) | 0.330 |  |  |
| **Diagnostic criteria** | | | | | | | |
| **mPAP (rest)** |  |  |  |  |  | <0.01 | 1.00 |
| other | 5 | 0.90 | 0.00 | 4.99 (4.38-5.60) | <0.001 |  |  |
| Slope-based | 3 | 0.12 | 52.88 | 3.75 (0.71-6.79) | <0.001 |  |  |
| **mPAP (exercise)** |  |  |  |  |  | 0.01 | 0.55 |
| other | 5 | 0.19 | 35.07 | 11.62 (9.94-13.30) | <0.001 |  |  |
| Slope-based | 3 | 0.15 | 48.12 | 8.19 (6.24-10.14) | <0.001 |  |  |
| **CO (Rest)** |  |  |  |  |  | 0.93 | 0.00 |
| other | 8 | <0.01 | 87.68 | -0.24 (-0.53, 0.05) | 0.105 |  |  |
| Slope-based | 3 | 0.30 | 17.90 | -0.21 (-0.72, 0.30) | 0.416 |  |  |
| **Disease condition** | | | | | | | |
| **mPAP (rest)** |  |  |  |  |  | 0.56 | 0.00 |
| Dyspnea | 2 | <0.01 | 96.29 | 2.71 (-0.82, 6.23) | 0.13 |  |  |
| CTS | 5 | 0.01 | 72.52 | 3.99 (1.59, 6.38) | <0.001 |  |  |
| **mPAP (exercise)** |  |  |  |  |  | 0.28 | 0.00 |
| Dyspnea | 2 | <0.01 | 88.89 | 8.62 (4.49, 12.76) | <0.001 |  |  |
| CTS | 5 | 0.01 | 69.74 | 11.42 (8.50, 14.34) | <0.001 |  |  |
| **CO (rest)** |  |  |  |  |  | 0.74 | 0.00 |
| Dyspnea | 3 | <0.01 | 94.69 | -0.41 (-0.90, 0.08) | 0.10 |  |  |
| CTS | 6 | 0.08 | 48.96 | -0.30 (-0.71, 0.12) | 0.16 |  |  |
| **CO (exercise)** |  |  |  |  |  | 0.58 | 0.00 |
| Dyspnea | 3 | <0.01 | 99.06 | -2.35 (-4.63, -0.07) | 0.04 |  |  |
| CTS | 6 | <0.01 | 80.25 | -1.55 (-3.28, 0.19) | 0.08 |  |  |
| **PVR (Rest)** |  |  |  |  |  | 0.58 | 0.00 |
| Apparently healthy | 2 | 0.03 | 79.89 | 0.49 (-0.11, 1.10) | 0.11 |  |  |
| CTS | 5 | <0.01 | 76.58 | 0.70 (0.28, 1.12) | 0.00 |  |  |
| **LVEF (Rest)** |  |  |  |  |  | 0.52 | 0.00 |
| CVD | 6 | 0.07 | 50.21 | 0.32 (-1.01, 1.64) | 0.64 |  |  |
| Dyspnea | 4 | <0.01 | 87.91 | 0.09 (-1.22, 1.40) | 0.89 |  |  |
| CTS | 5 | 0.70 | 0.00 | 1.19 (-0.27, 2.65) | 0.11 |  |  |
| **E/e'(rest)** |  |  |  |  |  | 0.05 | 0.00 |
| CVD | 5 | 0.02 | 65.27 | 2.78 (1.35, 4.20) | 0.00 |  |  |
| Dyspnea | 3 | <0.01 | 94.67 | 1.27 (-0.28, 2.81) | 0.11 |  |  |
| CTS | 5 | <0.01 | 85.21 | 0.41 (-0.86, 1.68) | 0.52 |  |  |
| **E/e' (exercise)** |  |  |  |  |  | 0.64 | 0.00 |
| CVD | 4 | <0.01 | 76.64 | 3.16 (1.37, 4.94) | 0.00 |  |  |
| CTS | 2 | <0.01 | 91.44 | 2.48 (0.30, 4.65) | 0.03 |  |  |
| **e' (rest)** |  |  |  |  |  | 0.77 | 0.00 |
| CVD | 4 | <0.01 | 81.12 | -0.85 (-1.63, -0.07) | 0.03 |  |  |
| Dyspnea | 2 | 0.00 | 93.34 | -1.07 (-2.07, -0.08) | 0.04 |  |  |
| CTS | 3 | 0.75 | 0.00 | -1.31 (-2.32, -0.31) | 0.01 |  |  |
| **E/A (Rest)** |  |  |  |  |  | 0.39 | 0.00 |
| CVD | 2 | 0.70 | 0.00 | 0.06 (-0.13, 0.25) | 0.54 |  |  |
| Dyspnea | 3 | 0.00 | 90.06 | -0.08 (-0.20, 0.05) | 0.22 |  |  |
| CTS | 3 | 0.60 | 0.00 | -0.11 (-0.26, 0.05) | 0.18 |  |  |
| **LVEDD** |  |  |  |  |  | 0.26 | 0.00 |
| CVD | 2 | 0.49 | 0.00 | 1.36 (-0.17, 2.89) | 0.08 |  |  |
| Dyspnea | 2 | 0.24 | 26.32 | 0.35 (-0.04, 074) | 0.08 |  |  |
| CTS | 2 | 0.82 | 0.00 | -0.08 (-0.91, 0.75 | 0.85 |  |  |
| **LVESD** |  |  |  |  |  | 0.50 | 0.00 |
| CVD | 2 | 0.54 | 0.00 | 0.15 (-1.42, 1.73) | 0.85 |  |  |
| Dyspnea | 2 | 0.06 | 72.64 | -0.45 (-1.22, 0.31) | 0.25 |  |  |
| **Body position during exercise** | | | | | | | |
| **mPAP(exercise)** |  |  |  |  |  | 0.99 | 0.00 |
| Supine | 4 | 0.00 | 77.91 | 10.00 (7.02, 12.99) | <0.001 |  |  |
| Upright | 2 | 0.02 | 81.34 | 9.98 (6.94, 13.02) | <0.001 |  |  |
| **CO (Exercise)** |  |  |  |  |  | 0.77 | 0.00 |
| Semi-supine | 3 | 0.78 | 0.00 | -0.94 (-3.54, 1.67) | 0.48 |  |  |
| Supine | 5 | <0.001 | 94.47 | -2.14 (-4.26, -0.02) | 0.05 |  |  |
| Upright | 2 | <0.001 | 99.48 | -1.90 (-5.00, 1.21) | 0.23 |  |  |
| **PVR (Exercise)** |  |  |  |  |  | NA | NA |
| Supine | 3 | 0.37 | 5.20 | 1.47 (1.19, 1.70) | <0.001 |  |  |
| **e' (Exercise)** |  |  |  |  |  | NA | NA |
| Semi-supine | 4 | <0.001 | 93.29 | -1.27 (-3.52, 0.97) | 0.27 |  |  |
| **E/e' (exercise)** |  |  |  |  |  | 0.49 | 0.00 |
| Semi-supine | 4 | 0.00 | 77.16 | 3.20 (1.37, 5.03) | <0.001 |  |  |
| Upright | 2 | <0.001 | 90.19 | 2.16 (-0.16, 4.47) | 0.07 |  |  |
| **LVEF (Exercise)** |  |  |  |  |  |  |  |
| Semi-supine | 5 | 0.92 | 0.00 | -0.49 (-2.01, 1.04) | 0.53 | <0.001 | 100.00 |
| **Exercise Intensity** | | | | | | | |
| **mPAP (Exercise)** |  |  |  |  |  | NA | NA |
| Symptom-limited | 7 | 0.01 | 65.74 | 10.55 (8.52, 12.57) | <0.001 |  |  |
| **CO (Exercise)** |  |  |  |  |  | NA | NA |
| Symptom-limited | 10 | <0.001 | 96.87 | -1.68 (-2.77, -0.59) | 0.002 |  |  |
| **PVR (Exercise)** |  |  |  |  |  | NA | NA |
| Symptom-limited | 6 | 0.52 | 0 | 1.40 (1.21, 1.59) | <0.001 |  |  |
| **E/e' (exercise)** |  |  |  |  |  | NA | NA |
| Symptom-limited | 7 | <0.001 | 88.47 | 2.68 (1.57, 3.79) | <0.001 |  |  |

**Note: For subgroups with only one study, no between-group comparison was possible.**

| **Supplementary Table 3. Meta-regression analysis of age** | | | | | | | |
| --- | --- | --- | --- | --- | --- | --- | --- |
| **Outcome** | **Time point** | **Number of Available Studies** | **Coefficient** | **95% Lower** | **95% Upper** | **P-Value** | **R2** |
| mPAP | rest | 8 | -0.32 | -0.57 | -0.07 | 0.01 | 0.54 |
|  | exercise | 8 | -0.53 | -0.78 | -0.28 | <0.01 | 1.00 |
| PVR | rest | 8 | -0.03 | -0.07 | -0.02 | 0.24 | 0.00 |
|  | exercise | 7 | -0.02 | -0.13 | 0.08 | 0.67 | 0.00 |
| CO | rest | 11 | 0.05 | -0.02 | 0.11 | 0.14 | 0.00 |
|  | exercise | 11 | 0.02 | -0.21 | 0.25 | 0.86 | 0.00 |
| LVEF | rest | 16 | 0.08 | -0.05 | 0.22 | 0.22 | 0.00 |
|  | exercise | 6 | 0.08 | -0.26 | 0.42 | 0.65 | 0.06 |
| E/e’ | rest | 14 | -0.07 | -0.20 | 0.05 | 0.26 | 0.13 |
|  | exercise | 8 | -0.07 | -0.21 | 0.07 | 0.32 | 0.17 |
| e' | rest | 10 | 0.07 | -0.01 | 0.14 | 0.09 | 0.03 |
|  | exercise | 6 | 0.21 | 0.00 | 0.42 | 0.06 | 0.00 |
| E/A | rest | 8 | 0.01 | 0.00 | 0.02 | 0.15 | 0.30 |
|  | exercise | NA | NA | NA | NA | NA | NA |
| LVEDD | rest | 7 | 0.15 | 0.00 | 0.29 | 0.05 | 0.00 |
| LVESD | rest | 6 | 0.18 | 0.04 | 0.32 | 0.01 | 1.00 |

| **Supplementary Table 4. Meta-regression analysis of female patients** | | | | | | | |
| --- | --- | --- | --- | --- | --- | --- | --- |
| **Outcome** | **Time point** | **Number of Available Studies** | **Coefficient** | **95% Lower** | **95% Upper** | **P-Value** | **R2** |
| mPAP | rest | 8 | 0.012 | -0.083 | 0.107 | 0.80 | 0.00 |
|  | exercise | 8 | 0.049 | -0.073 | 0.172 | 0.43 | 0.00 |
| PVR | rest | 8 | 0.002 | -0.019 | 0.015 | 0.83 | 0.00 |
|  | exercise | 7 | -0.002 | -0.030 | 0.026 | 0.88 | 0.08 |
| CO | rest | 11 | -0.002 | -0.017 | 0.012 | 0.77 | 0.00 |
|  | exercise | 11 | 0.003 | -0.053 | 0.058 | 0.93 | 0.00 |
| LVEF | rest | 16 | 0.017 | -0.017 | 0.050 | 0.33 | 0.00 |
|  | exercise | 6 | -0.023 | -0.152 | 0.106 | 0.73 | 0.00 |
| e' | rest | 10 | -0.007 | -0.027 | 0.013 | 0.47 | 0.00 |
|  | exercise | 6 | -0.065 | -0.158 | 0.029 | 0.18 | 0.00 |
| E/e’ | rest | 14 | -0.042 | -0.074 | -0.010 | 0.01 | 0.00 |
|  | exercise | 8 | -0.021 | -0.074 | 0.032 | 0.44 | 0.00 |
| E/A | rest | 8 | -0.003 | -0.007 | 0.001 | 0.13 | 0.26 |
|  | exercise | NA | NA | NA | NA | NA | NA |
| LVEDD | rest | 7 | -0.019 | -0.042 | 0.004 | 0.11 | 0.00 |
| LVESD | rest | 6 | -0.029 | -0.051 | -0.008 | 0.01 | 1.00 |

**Supplementary Fig. 1. Bubble plot of the meta-regression of mean difference in mPAP on age at rest.**

**Supplementary Fig. 2. Bubble plot of the meta-regression of mean difference in mPAP on age at exercise.**

**Supplementary Fig. 3. Bubble plot of the meta-regression of mean difference in LVEDD on age at rest.**

**Supplementary Fig. 4. Bubble plot of the meta-regression of mean difference in LVESD on age at rest.**

**Supplementary Fig. 5. Bubble plot of the meta-regression of mean difference in E/e’ on %female at rest.**

**Supplementary Fig. 6. Bubble plot of the meta-regression of mean difference in LVESD on %female at rest.**

**Supplementary Table 5. Sensitivity analyses (one-study remove analyses)**

| **Study name** | **Time-point** | **Statistics with study removed** | | | |
| --- | --- | --- | --- | --- | --- |
|  |  | **MD** | **Lower limit** | **Upper limit** | **p-Value** |
| **mPAP** | | | | | |
| Voilliot, D. (2014) | Rest | 3.41 | 1.85 | 4.97 | <0.01 |
| Ho, J. E. (2020) | Rest | 3.46 | 1.90 | 5.02 | <0.01 |
| Saggar, R.(2010) | Rest | 3.55 | 1.95 | 5.16 | <0.01 |
| Tolle, J. J. (2008) | Rest | 3.56 | 1.90 | 5.22 | <0.01 |
| Miyanaga, S.(2019) | Rest | 3.64 | 2.05 | 5.23 | <0.01 |
| Kanazawa, Y. (2024) | Rest | 3.78 | 2.21 | 5.36 | <0.01 |
| Kusunose, K. (2019) | Rest | 4.01 | 2.47 | 5.55 | <0.01 |
| Skjørten, I. (2018) | Rest | 4.23 | 2.87 | 5.59 | <0.01 |
| Overall |  | 3.70 | 2.24 | 5.17 | <0.01 |
| Saggar, R.(2010) | Exercise | 9.76 | 8.16 | 11.35 | <0.01 |
| Voilliot, D. (2014) | Exercise | 9.85 | 8.17 | 11.52 | <0.01 |
| Miyanaga, S.(2019) | Exercise | 10.07 | 8.44 | 11.71 | <0.01 |
| Ho, J. E. (2020) | Exercise | 10.10 | 8.05 | 12.15 | <0.01 |
| Kanazawa, Y. (2024) | Exercise | 10.37 | 8.60 | 12.14 | <0.01 |
| Tolle, J. J. (2008) | Exercise | 10.40 | 8.56 | 12.24 | <0.01 |
| Kusunose, K. (2019) | Exercise | 10.55 | 8.52 | 12.57 | <0.01 |
| Skjørten, I. (2018) | Exercise | 10.76 | 9.24 | 12.27 | <0.01 |
| Overall |  | 10.23 | 8.60 | 11.85 | <0.01 |
| **PVR** | | | | | |
| Voilliot, D. (2014) | Rest | 0.41 | 0.26 | 0.56 | <0.01 |
| Saggar, R.(2010) | Rest | 0.46 | 0.29 | 0.64 | <0.01 |
| Tolle, J. J. (2008) | Rest | 0.47 | 0.29 | 0.65 | <0.01 |
| Miyanaga, S.(2019) | Rest | 0.48 | 0.30 | 0.66 | <0.01 |
| Skjørten, I. (2018) | Rest | 0.52 | 0.32 | 0.71 | <0.01 |
| Kanazawa, Y. (2024) | Rest | 0.52 | 0.34 | 0.71 | <0.01 |
| Suzuki, K. (2014) | Rest | 0.67 | 0.35 | 0.99 | <0.01 |
| Shim, C. Y. (2011) | Rest | 0.68 | 0.38 | 0.98 | <0.01 |
| Overall |  | 0.51 | 0.33 | 0.69 | <0.01 |
| Saggar, R.(2010) | Exercise | 1.22 | 0.74 | 1.70 | <0.01 |
| Miyanaga, S.(2019) | Exercise | 1.24 | 0.74 | 1.74 | <0.01 |
| Voilliot, D. (2014) | Exercise | 1.26 | 0.75 | 1.77 | <0.01 |
| Kanazawa, Y. (2024) | Exercise | 1.30 | 0.77 | 1.83 | <0.01 |
| Skjørten, I. (2018) | Exercise | 1.30 | 0.76 | 1.85 | <0.01 |
| Tolle, J. J. (2008) | Exercise | 1.31 | 0.76 | 1.86 | <0.01 |
| Suzuki, K. (2014) | Exercise | 1.40 | 1.21 | 1.59 | <0.01 |
| Overall |  | 1.30 | 0.82 | 1.78 | <0.01 |
| **CO** | | | | | |
| Shim, C. Y. (2011) | Rest | -0.28 | -0.55 | -0.01 | 0.05 |
| Magne, J. (2011) | Rest | -0.28 | -0.52 | -0.03 | 0.03 |
| Suzuki, K. (2014) | Rest | -0.26 | -0.54 | 0.01 | 0.06 |
| Kanazawa, Y. (2024) | Rest | -0.26 | -0.51 | -0.01 | 0.04 |
| Lim, A. Y. (2017) | Rest | -0.26 | -0.56 | 0.05 | 0.10 |
| Kusunose, K. (2019) | Rest | -0.22 | -0.49 | 0.04 | 0.10 |
| Voilliot, D. (2014) | Rest | -0.22 | -0.47 | 0.04 | 0.10 |
| Skjørten, I. (2018) | Rest | -0.21 | -0.47 | 0.05 | 0.11 |
| Miyanaga, S.(2019) | Rest | -0.21 | -0.46 | 0.04 | 0.10 |
| Saggar, R.(2010) | Rest | -0.19 | -0.43 | 0.06 | 0.13 |
| Ho, J. E. (2020) | Rest | -0.11 | -0.28 | 0.05 | 0.18 |
| Overall |  | -0.23 | -0.47 | 0.01 | 0.06 |
| Shim, C. Y. (2011) | Exercise | -1.70 | -2.83 | -0.57 | <0.01 |
| Suzuki, K. (2014) | Exercise | -1.68 | -2.77 | -0.59 | <0.01 |
| Lim, A. Y. (2017) | Exercise | -1.68 | -2.85 | -0.51 | <0.01 |
| Magne, J. (2011) | Exercise | -1.59 | -2.60 | -0.58 | <0.01 |
| Kusunose, K. (2019) | Exercise | -1.56 | -2.60 | -0.52 | <0.01 |
| Voilliot, D. (2014) | Exercise | -1.53 | -2.55 | -0.52 | <0.01 |
| Kanazawa, Y. (2024) | Exercise | -1.48 | -2.47 | -0.48 | <0.01 |
| Miyanaga, S.(2019) | Exercise | -1.45 | -2.42 | -0.47 | <0.01 |
| Saggar, R.(2010) | Exercise | -1.33 | -2.31 | -0.34 | 0.01 |
| Skjørten, I. (2018) | Exercise | -1.30 | -2.27 | -0.33 | 0.01 |
| Ho, J. E. (2020) | Exercise | -1.09 | -1.71 | -0.47 | <0.01 |
| Overall |  | -1.50 | -2.45 | -0.54 | <0.01 |
| **e’** | | | | | |
| Lancellotti, P. (2012) | Rest | -1.09 | -1.44 | -0.74 | <0.01 |
| Lim, A. Y. (2017) | Rest | -1.06 | -1.45 | -0.66 | <0.01 |
| Shim, C. Y. (2011) | Rest | -1.03 | -1.46 | -0.60 | <0.01 |
| Amano, M. (2022) | Rest | -0.97 | -1.36 | -0.57 | <0.01 |
| Kanazawa, Y. (2024) | Rest | -0.96 | -1.35 | -0.57 | <0.01 |
| Suzuki, K. (2014) | Rest | -0.96 | -1.38 | -0.54 | <0.01 |
| Magne, J. (2011) | Rest | -0.94 | -1.34 | -0.55 | <0.01 |
| Voilliot, D. (2014) | Rest | -0.94 | -1.31 | -0.57 | <0.01 |
| Lancellotti, P. (2015) | Rest | -0.93 | -1.33 | -0.53 | <0.01 |
| Ha, J. W. (2009) | Rest | -0.86 | -1.20 | -0.52 | <0.01 |
| Overall |  | -0.99 | -1.49 | -0.50 | <0.01 |
| Shim, C. Y. (2011) | Exercise | -1.01 | -2.53 | 0.51 | 0.19 |
| Lim, A. Y. (2017) | Exercise | -1.15 | -2.53 | 0.23 | 0.10 |
| Lancellotti, P. (2012) | Exercise | -1.42 | -2.37 | -0.47 | <0.01 |
| Magne, J. (2011) | Exercise | -0.97 | -2.16 | 0.22 | 0.11 |
| Lancellotti, P. (2015) | Exercise | -0.71 | -1.89 | 0.47 | 0.24 |
| Voilliot, D. (2014) | Exercise | -0.50 | -1.53 | 0.53 | 0.34 |
| Overall |  | -0.86 | -1.55 | -1.55 | 0.01 |
| **E/e’** | | | | | |
| Magne, J. (2011) | Rest | 1.30 | 0.63 | 1.97 | <0.01 |
| Ha, J. W. (2009) | Rest | 1.32 | 0.65 | 1.98 | <0.01 |
| Re, F. (2022) | Rest | 1.38 | 0.68 | 2.08 | <0.01 |
| Shim, C. Y. (2011) | Rest | 1.39 | 0.66 | 2.12 | <0.01 |
| Magne, J. (2015) | Rest | 1.40 | 0.70 | 2.10 | <0.01 |
| Lancellotti, P. (2015) | Rest | 1.43 | 0.72 | 2.13 | <0.01 |
| Suzuki, K. (2014) | Rest | 1.49 | 0.71 | 2.26 | <0.01 |
| Voilliot, D. (2014) | Rest | 1.50 | 0.77 | 2.23 | <0.01 |
| Kanazawa, Y. (2024) | Rest | 1.53 | 0.82 | 2.24 | <0.01 |
| Kusunose, K. (2019) | Rest | 1.53 | 0.78 | 2.28 | <0.01 |
| Lancellotti, P. (2012) | Rest | 1.53 | 0.81 | 2.25 | <0.01 |
| Shim, C. Y. (2011) | Rest | 1.54 | 0.72 | 2.36 | <0.01 |
| Skjørten, I. (2018) | Rest | 1.64 | 0.95 | 2.33 | <0.01 |
| Miyanaga, S.(2019) | Rest | 1.75 | 1.14 | 2.36 | <0.01 |
| Overall |  | 1.48 | 0.80 | 2.16 | <0.01 |
| Lancellotti, P. (2015) | Exercise | 2.19 | 1.35 | 3.03 | <0.01 |
| Suzuki, K. (2014) | Exercise | 2.24 | 1.37 | 3.11 | <0.01 |
| Re, F. (2022) | Exercise | 2.32 | 1.40 | 3.24 | <0.01 |
| Magne, J. (2011) | Exercise | 2.34 | 1.42 | 3.26 | <0.01 |
| Shim, C. Y. (2011) | Exercise | 2.52 | 1.46 | 3.58 | <0.01 |
| Suzuki, K. (2014) | Exercise | 2.68 | 1.57 | 3.79 | <0.01 |
| Lancellotti, P. (2012) | Exercise | 2.69 | 1.76 | 3.62 | <0.01 |
| Lim, A. Y. (2017) | Exercise | 2.73 | 1.78 | 3.68 | <0.01 |
| Overall |  | 2.46 | 1.57 | 3.34 | <0.01 |
| **LVEF** | | | | | |
| Ha, J. W. (2009) | Rest | 0.43 | -0.22 | 1.09 | 0.19 |
| Lim, A. Y. (2017) | Rest | 0.47 | -0.25 | 1.18 | 0.20 |
| Amano, M. (2022) | Rest | 0.47 | -0.20 | 1.15 | 0.17 |
| Voilliot, D. (2014) | Rest | 0.48 | -0.14 | 1.10 | 0.13 |
| Kusunose, K. (2019) | Rest | 0.49 | -0.21 | 1.18 | 0.17 |
| Shim, C. Y. (2011) | Rest | 0.50 | -0.18 | 1.19 | 0.15 |
| Lancellotti, P. (2012) | Rest | 0.51 | -0.13 | 1.15 | 0.12 |
| Lancellotti (2015) | Rest | 0.52 | -0.13 | 1.17 | 0.11 |
| Kanazawa, Y. (2024) | Rest | 0.53 | -0.11 | 1.17 | 0.11 |
| Miyanaga, S.(2019) | Rest | 0.53 | -0.09 | 1.16 | 0.09 |
| Suzuki, K. (2014) | Rest | 0.54 | -0.14 | 1.21 | 0.12 |
| Magne, J. (2015) | Rest | 0.54 | -0.10 | 1.18 | 0.10 |
| Magne, J. (2011) | Rest | 0.57 | -0.07 | 1.21 | 0.08 |
| Skjørten, I. (2018) | Rest | 0.64 | 0.01 | 1.26 | 0.05 |
| Re, F. (2022) | Rest | 0.70 | 0.14 | 1.26 | 0.01 |
| Ho, J. E. (2020) | Rest | 0.88 | 0.45 | 1.32 | <0.01 |
| Overall |  | 0.55 | -0.07 | 1.17 | 0.08 |
| Magne, J. (2011) | Exercise | -1.75 | -3.70 | 0.20 | 0.08 |
| Voilliot, D. (2014) | Exercise | -1.61 | -3.70 | 0.48 | 0.13 |
| Lancellotti (2015) | Exercise | -1.36 | -3.62 | 0.90 | 0.24 |
| Magne, J. (2015) | Exercise | -1.36 | -3.61 | 0.89 | 0.24 |
| Lancellotti, P. (2012) | Exercise | -1.36 | -3.59 | 0.87 | 0.23 |
| Ho, J. E. (2020) | Exercise | -0.49 | -2.01 | 1.03 | 0.53 |
| Overall |  | -1.96 | -3.25 | -0.69 | 0.003 |
| **E/A** | | | | | |
| Magne, J. (2015) | Rest | -0.07 | -0.15 | 0.01 | 0.10 |
| Lancellotti (2015) | Rest | -0.07 | -0.16 | 0.01 | 0.10 |
| Lim, A. Y. (2017) | Rest | -0.08 | -0.16 | 0.01 | 0.08 |
| Voilliot, D. (2014) | Rest | -0.06 | -0.15 | 0.02 | 0.14 |
| Skjørten, I. (2018) | Rest | -0.06 | -0.16 | 0.03 | 0.17 |
| Suzuki, K. (2014) | Rest | -0.05 | -0.14 | 0.04 | 0.27 |
| Kanazawa, Y. (2024) | Rest | -0.05 | -0.13 | 0.04 | 0.28 |
| Ha, J. W. (2009) | Rest | -0.02 | -0.07 | 0.03 | 0.37 |
| **Overall** |  | -0.06 | -0.14 | 0.02 | 0.14 |
| Magne, J. (2015) | Exercise | -0.01 | -0.11 | 0.10 | 0.90 |
| Lancellotti (2015) | Exercise | -0.01 | -0.10 | 0.08 | 0.89 |
| Lim, A. Y. (2017) | Exercise | 0.04 | -0.14 | 0.22 | 0.66 |
| Voilliot, D. (2014) | Exercise | 0.05 | -0.04 | 0.14 | 0.26 |
| Overall |  | 0.02 | -0.09 | 0.13 | 0.72 |
| **LVEDD** | | | | | |
| Lim, A. Y. (2017) | Rest | 0.08 | -0.33 | 0.48 | 0.71 |
| Magne, J. (2015) | Rest | 0.23 | -0.08 | 0.54 | 0.15 |
| Amano, M. (2022) | Rest | 0.24 | -0.07 | 0.55 | 0.12 |
| Miyanaga, S.(2019) | Rest | 0.25 | -0.07 | 0.57 | 0.13 |
| Suzuki, K. (2014) | Rest | 0.31 | -0.01 | 0.64 | 0.06 |
| Ha, J. W. (2009) | Rest | 0.32 | -0.02 | 0.66 | 0.07 |
| Shim, C. Y. (2011) | Rest | 0.32 | -0.02 | 0.67 | 0.06 |
| Overall |  | 0.24 | -0.16 | 0.64 | 0.24 |
| **LVESD** |  |  |  |  |  |
| Lim, A. Y. (2017) | Rest | -0.83 | -1.24 | -0.41 | <0.01 |
| Amano, M. (2022) | Rest | -0.61 | -1.15 | -0.08 | 0.03 |
| Magne, J. (2015) | Rest | -0.60 | -1.10 | -0.11 | 0.02 |
| Shim, C. Y. (2011) | Rest | -0.56 | -1.19 | 0.07 | 0.08 |
| Ha, J. W. (2009) | Rest | -0.47 | -1.01 | 0.07 | 0.09 |
| Suzuki, K. (2014) | Rest | -0.38 | -0.79 | 0.03 | 0.07 |
| Overall |  | -0.54 | -0.94 | -0.15 | 0.01 |

**Supplementary Table 6. Sensitivity analyses restricted to studies with NOS ≥ 7**

| subgroup | No. of available study | Heterogeneity | | Pooled Effect Size | |
| --- | --- | --- | --- | --- | --- |
|  |  | P-Value | I² | MD (95% CI) | P-Value |
| **mPAP (Rest)** |  |  |  |  |  |
| ≥7 | 2 | 0.89 | 0 | 4.79 (1.74, 7.85) | <0.01 |
| **mPAP (Exercise)** |  |  |  |  |  |
| ≥7 | 2 | 0.04 | 75.58 | 11.35 (8.00, 14.71) | <0.01 |
| **CO (Rest)** |  |  |  |  |  |
| ≥7 | 4 | 0.03 | 66.57 | -0.03 (-0.45, 0.39) | 0.88 |
| **CO (Exercise)** |  |  |  |  |  |
| ≥7 | 4 | <0.01 | 84.52 | -0.86 (-2.47, 0.75) | 0.29 |
| **PVR (Rest)** |  |  |  |  |  |
| ≥7 | 4 | 0.02 | 71.18 | 0.38 (0.21, 0.55) | <0.01 |
| **PVR (Exercise)** |  |  |  |  |  |
| ≥7 | 3 | <0.01 | 94.42 | 1.12 (0.51, 1.72) | <0.01 |
| **e' (Rest)** |  |  |  |  |  |
| ≥7 | 5 | 0.02 | 64.6 | -1.19 (-1.61, -0.77) | <0.01 |
| **e' (Exercise)** |  |  |  |  |  |
| ≥7 | 3 | 0.03 | 71.27 | -1.30 (-2.85, 0.25) | 0.1 |
| **E/e' (Rest)** |  |  |  |  |  |
| ≥7 | 6 | <0.01 | 73.2 | 2.71 (1.84, 3.58) | <0.01 |
| **E/e' (Exercise)** |  |  |  |  |  |
| ≥7 | 5 | <0.01 | 76.61 | 2.99 (1.73, 4.26) | <0.01 |
| **LVEF (Rest)** |  |  |  |  |  |
| ≥7 | 6 | 0.04 | 57.52 | 0.51 (-0.51, 1.53) | 0.33 |
| **LVEF (Exercise)** |  |  |  |  |  |
| ≥7 | 2 | 0.44 | 0 | -0.13 (-3.51, 3.26) | 0.94 |
| **E/A (Rest)** |  |  |  |  |  |
| ≥7 | 2 | 0.04 | 77.04 | -0.15 (-0.27, -0.03) | 0.02 |
| **E/A (Exercise)** |  |  |  |  |  |
| ≥7 | 1 | NA | NA | 0.10 (-0.11, 0.31) | 0.35 |
| **LVEDD (Rest)** |  |  |  |  |  |
| ≥7 | 3 | 0.98 | 0 | -0.03 (-0.45, 0.40) | 0.91 |
| **LVESD (Rest)** |  |  |  |  |  |
| ≥7 | 3 | 0.5 | 0 | -0.92 (-1.35, -0.48) | <0.01 |
| **SV (Rest)** |  |  |  |  |  |
| ≥7 | 3 | 0.32 | 11.97 | 1.17 (-0.68, 3.03) | 0.21 |
| **SV (Exercise)** |  |  |  |  |  |
| ≥7 | 2 | 0.39 | 0 | -1.62 (-4.32, 1.08) | 0.24 |

**Funnel Plot**

**Supplementary Fig. 7. mPAP (REST)**

Egger's regression p-value = 0.939

**Supplementary Fig. 8. mPAP (EXERCISE)**

Egger's regression p-value = 0.558

**Supplementary Fig. 9. PVR (REST)**

Egger's regression p-value = 0.012

PVR (Rest): trimmed 4 studies; 0.31 (0.12, 0.50)

**Supplementary Fig. 10. PVR (EXERCISE)**

Egger's regression p-value < 0.001

PVR (Exercise): trimmed 3 studies; 1.14 (0.75, 1.53)

**Supplementary Fig. 11. CO (REST)**

Egger's regression p-value= 0.518

**Supplementary Fig. 12. CO (EXERCISE)**

Egger's regression p-value= 0.210

**Supplementary Fig. 13. e’ (REST)**

Egger's regression p-value= 0.25

**Supplementary Fig. 14. e’ (EXERCISE)**

Egger's regression p-value= 0.830

**Supplementary Fig. 15. E/e’ (REST)**

Egger's regression p-value = 0.669

**Supplementary Fig. 16. E/e’ (EXERCISE)**

Egger's regression p-value = 0.047

E/e' (Exercise): trimmed 3 studies; 1.57 (0.67, 2.47)

**Supplementary Fig. 17. LVEF (REST)**

Egger's regression p-value = 0.465

**Supplementary Fig. 18. LVEF (EXERCISE)**

Egger's regression p-value < 0.001

LVEF (Exercise): trimmed 3 studies; 0.32 (-0.30, 0.95)

**Supplementary Fig. 19. E/A (REST)**

Egger's regression p-value = 0.721

**Supplementary Fig. 20. E/A (EXERCISE)**

Egger's regression p-value = 0.740

**Supplementary Fig. 21. LVEDD (REST)**

Egger's regression p-value = 0.560

**Supplementary Fig. 22. LVESD (REST)**

Egger's regression p-value = 0.740

**Supplementary Fig. 23. SV(REST)**

Egger's regression p-value = 0.290

**Supplementary Fig. 24. SV(EXERCISE)**

Egger's regression p-value = 0.137
